# External factors show reproducible local symptom-biomarker associations in middle-aged and older adults with heart disease

**DOI:** 10.3389/fpsyt.2026.1870992

**Published:** 2026-06-02

**Authors:** Haoke Shi, Lihua Yang, Yijie Fang, Hongping Lu, Yangyang Huang, Zhiyong Xiao, Yongxin Long, Peng Li, Fengzhi Shi, Hongwu Liao, Xinhong Yin

**Affiliations:** 1 School of Nursing, University of South China, Hengyang, Hunan, China; 2 Affiliated Hospital of Xiangnan University, Chenzhou, Hunan, China; 3 Health School of Nuclear Industry, Hengyang, Hunan, China; 4 Department of Pain Medicine, Hubei Provincial Hospital of Traditional Chinese Medicine, Wuhan, Hubei, China; 5 School of Horticulture, Hunan Agricultural University, Changsha, Hunan, China; 6 Department of Gastroenterology, Affiliated Nanhua Hospital, Hengyang Medical College, University of South China, Hengyang, Hunan, China

**Keywords:** depression, heart disease, biomarkers, multimorbidity, caregiving status, sex, network analysis, external replication

## Abstract

**Background:**

Depression in middle-aged and older adults with heart disease is clinically heterogeneous, but this heterogeneity is often assessed using total symptom scores or symptom-only approaches. We examined whether multimorbidity burden, caregiving status, and sex were associated with a joint depressive symptom-biomarker network and whether the main patterns were reproducible in an independent hospital cohort.

**Methods:**

We analyzed 1,685 participants with heart disease from the China Health and Retirement Longitudinal Study as a discovery cohort and 506 patients from an independent hospital cohort for external replication. We estimated a joint depressive symptom-biomarker network and examined node-specific associations of the three external factors, as well as subgroup differences in network structure and global connectivity.

**Results:**

The external factors were associated with heterogeneous local patterns in the joint symptom-biomarker network, whereas significant subgroup differences in overall network structure or global connectivity were not observed. The factor-specific patterns were broadest across domains for multi-morbidity burden, weaker and more localized for caregiving status, and clearest and most reproducible for sex, particularly involving high-density lipoprotein cholesterol, triglycerides, and cystatin C. Across both cohorts, depressed mood consistently showed the highest centrality among symptom nodes.

**Conclusions:**

In middle-aged and older adults with heart disease, depressive heterogeneity was reflected more clearly in reproducible local symptom-biomarker associations than in broad network-wide differences. These findings support an assessment-oriented approach that integrates external factors and routinely available biomarkers when characterizing depressive heterogeneity in this population.

## 1 Introduction

Cardiovascular disease (CVD) and depression are major public health problems that frequently co-occur (1). Previous research has shown a bidirectional relationship between CVD and depression: individuals with CVD are at increased risk of developing depression, whereas those with depression are also more likely to experience subsequent CVD (2–4). This comorbidity involves interacting behavioral, functional, and biological pathways that may amplify adverse health risks (5). Compared with patients with CVD alone, those with co-morbid depression have an approximately threefold higher risk of mortality, underscoring the clinical importance of this condition (6). In middle-aged and older adults with heart disease, depression is also markedly heterogeneous (7). Patients with similar overall severity may show different symptom profiles. This heterogeneity may reflect sex, disease burden, physiological dysregulation, and social context, including chronic comorbidity, inflammation-related and metabolic processes, and caregiving context (7–9). Together, these findings suggest that total depression scores may not adequately capture this complexity and that a symptom-level approach may be more appropriate (10).

The traditional latent variable paradigm conceptualizes depression as a single underlying construct, with correlations among questionnaire items regarded as manifestations of that construct (11, 12). However, this framework has limited explanatory value in the context of marked depressive heterogeneity (13, 14). This limitation is particularly relevant in middle-aged and older adults with heart disease, in whom somatic and motivational symptoms are often closely related to disease burden and functional status. As a result, reliance on total scores alone may obscure differences in symptom configurations (15, 16). By contrast, network theory conceptualizes depression as a system of interacting symptoms and may therefore provide a more suitable framework for identifying key symptoms and structural features of depressive phenotypes in this population (17). Yet symptom-only networks may still be insufficient in patients with heart disease, because CVD-depression comorbidity involves inflammation, dysregulation of glucose and lipid metabolism, and impaired cardiac and renal function, and these processes may relate differently to specific symptoms. Prior studies suggest symptom-specific links, especially for fatigue, sleep disturbance, and reduced activity (18–20). A joint depressive symptom-biomarker network may therefore help identify key cross-domain nodes and edges while clarifying links between symptoms and physiological abnormalities (21, 22). The selected biomarkers were intended to capture several clinically relevant physiological domains, including peripheral inflammation, metabolic abnormalities, and cardiorenal/hemodynamic risk states, thereby providing a clinically relevant, though not exhaustive, window into these relationships.

Recent network studies have increasingly examined how external factors are associated with symptom systems. Recent network and prediction-modeling studies have further shown that depressive symptoms in older adults and cardiometabolic populations are embedded in broader cognitive, frailty-related, and cardiovascular contexts, supporting the need to examine depression heterogeneity beyond total symptom scores (15, 23, 24). In the present study, the term “external factors” is used descriptively to refer to variables outside the 19-node depressive symptom-biomarker network and does not imply temporal precedence or causal effects. Existing evidence suggests that these factors may be selectively associated with specific depressive symptoms or local network features rather than uniformly associated with the symptom system (25–27). However, in middle-aged and older adults with heart disease, direct evidence remains limited regarding how external factors are associated with a joint depressive symptom-biomarker network. In this population, three external factors are of particular clinical relevance: multi-morbidity burden, caregiving status, and sex (27, 28). In the present study, caregiving status was treated as a contextual indicator of caregiver presence versus absence rather than a direct measure of support adequacy. Previous studies have generally examined such factors either by estimating their conditional associations with specific symptoms or by comparing network characteristics across different external conditions (18, 25, 29). For example, somatic disease burden has been linked to direct bridging connections with specific depressive symptoms in older adults, suggesting that multi-morbidity may be associated with depressive phenotypes at the symptom level (30). At the same time, findings from single samples may be difficult to generalize across settings, particularly when symptom networks are embedded in different clinical and demographic contexts. In a population such as middle-aged and older adults with heart disease, replication in an independent clinical sample may therefore strengthen the interpretability and robustness of observed symptom-biomarker patterns.

In the present context, “heart disease” in CHARLS should be understood as a broad physician-diagnosed clinical category rather than a subtype-specific cardiovascular diagnosis. This category may encompass heterogeneous conditions, including coronary heart disease, heart failure, arrhythmias, and valvular disorders. These conditions differ in hemodynamic burden, inflammatory activity, cardiac function, symptom burden, and treatment exposure. Accordingly, any joint depressive symptom-biomarker network estimated in such a sample should be interpreted as capturing aggregate patterns across heterogeneous cardiac phenotypes rather than a single disease-specific mechanism.

The present study examined whether clinically relevant external factors were associated with factor-specific conditional patterns within a joint depressive symptom-biomarker network in middle-aged and older adults with heart disease. Using a population-based China Health and Retirement Longitudinal Study (CHARLS) discovery sample and an independent hospital cohort for external replication, we investigated the overall structure of the joint network, the conditional associations of multi-morbidity burden, caregiving status, and sex with specific symptom and biomarker nodes, and the reproducibility of the main findings across cohorts.

## 2 Methods

### 2.1 Study populations

#### 2.1.1 Discovery cohort: CHARLS

The discovery cohort was drawn from the China Health and Retirement Longitudinal Study (CHARLS), a nationally representative longitudinal survey of middle-aged and older adults in China (31). We used data from the 2015 follow-up wave (Wave 3). Participants were eligible if they reported a physician diagnosis of heart disease and had complete data on all 10 Center for Epidemiologic Studies Depression Scale items (CES-D-10), the nine biomarkers, and the three external factors. In CHARLS 2015, heart disease was identified using a self-reported physician-diagnosed chronic disease item and was therefore treated in the present study as a broad physician-diagnosed cardiovascular category rather than a subtype-specific diagnosis. Although CHARLS 2015 included limited disease-history information and some treatment-related items for certain chronic conditions, it did not provide sufficiently detailed cardiovascular phenotyping data, such as cardiovascular subtype, disease severity, cardiac function, ejection fraction, New York Heart Association functional class, or treatment information detailed enough for subtype- or severity-informed analyses. Therefore, subgroup analyses by cardiovascular diagnosis or adjustment for cardiac severity indicators could not be performed in the discovery cohort. Of 2,855 participants with physician-diagnosed heart disease identified in CHARLS 2015, 1,685 had complete data on all 10 CES-D-10 items, the nine biomarkers, and the three external factors and were included in the primary discovery analysis. The participant selection flow diagram and comparisons between included and excluded participants are presented in **Supplementary Table 1**.

CHARLS was approved by the Institutional Review Board of Peking University (main household survey, including physical measurements: IRB00001052-11015; biomarker collection: IRB00001052-11014), and all participants provided written informed consent. The present analysis used de-identified data.

#### 2.1.2 External validation cohort: independent hospital cohort

Key findings were externally replicated in an independent multicenter hospital cohort of middle-aged and older adults with heart disease. Participants were recruited consecutively from the First Affiliated Hospital of the University of South China, the Second Affiliated Hospital of the University of South China, and the Affiliated Nanhua Hospital of the University of South China between November 2025 and March 2026. Eligible participants were aged ≥45 years, had clinically confirmed heart disease, completed depressive symptom assessment, and had the biomarker and external factor data required for the replication analyses. Participants were excluded if they had missing depressive symptom items, missing key external factor data, or failed to meet other prespecified analytic conditions. The same depressive symptom measure, biomarker definitions, external factor definitions, and coding principles were used as in the discovery cohort. In the independent hospital cohort, missingness was limited and concentrated in two biomarkers: HbA1c was missing in 10 patients (2.0%) and CysC in 11 patients (2.2%). These missing values were handled using multiple imputation by chained equations, with an imputation model including all symptom nodes, biomarker variables, external factors, and available demographic variables (32). A total of 20 imputed datasets were generated. Complete-case analyses were additionally performed in the 485 patients with complete HbA1c and CysC data as a sensitivity check of the main validation network patterns. The final independent hospital cohort comprised 506 patients for the primary replication analyses.

This cohort was prespecified to assess whether the core nodes, strongest edges, and external factor-related patterns could be observed again, rather than to achieve exact replication of every parameter. The study protocol was approved by the Medical Ethics Committee of the University of South China (approval No. 2025-0121), and written informed consent was obtained from all participants.

### 2.2 Measures

#### 2.2.1 Depressive symptoms

Depressive symptoms were assessed using the 10-item Center for Epidemiologic Studies Depression Scale (CES-D-10), which has demonstrated good reliability and validity in Chinese older adults (33, 34). Each item was rated from 0 to 3, and positively worded items were reverse-coded. Total scores range from 0 to 30, with higher scores indicating greater depressive symptom burden. For descriptive purposes, a CES-D-10 score ≥10 was used to indicate elevated depressive symptoms (34, 35). Total scores were not used in the primary network estimation; instead, all 10 CES-D-10 items were entered separately as symptom nodes (A1-A6, B1-B4). Item wording and coding are provided in **Supplementary Table 2**.

#### 2.2.2 Biomarkers

Biomarker data were obtained from venous blood assessments collected in CHARLS Wave 3 (36). The selected biomarkers were chosen as a clinically interpretable set of routinely available markers that could capture several physiological domains implicated in depression-cardiovascular disease comorbidity and were available in both cohorts (1). Nine biomarkers were included as network nodes: body mass index (BMI), mean systolic blood pressure (SBP), white blood cell count (WBC), high-density lipoprotein cholesterol (HDL-C), fasting glucose (GLU), cystatin C (CysC), glycated hemoglobin (HbA1c), triglycerides (TG), and C-reactive protein (CRP).

The selection strategy was pathway-oriented rather than disease-specific. CRP and WBC indexed systemic inflammatory activity; GLU and HbA1c represented short- and longer-term glycemic dysregulation; TG and HDL-C represented lipid metabolism; SBP reflected hemodynamic and vascular burden; BMI reflected adiposity-related burden; and CysC indexed cardiorenal vulnerability (8, 37–42). Together, these biomarkers provided a clinically relevant but non-exhaustive representation of shared biological pathways that may connect depressive heterogeneity with cardiovascular risk states in middle-aged and older adults. They were not intended to constitute a disease-specific cardiovascular phenotyping panel. Biomarkers such as natriuretic peptides, troponins, and echocardiographic indices were not included because they were unavailable in CHARLS 2015 and were not comparable across the two cohorts. Detailed variable codes and descriptions are provided in **Supplementary Table 2**.

#### 2.2.3 External factors

Three external factors were examined: multi-morbidity burden, caregiving status, and sex. multi-morbidity burden was defined as the number of physician-diagnosed chronic conditions other than heart disease reported in CHARLS (43–45). In the present study, caregiving status was derived from the available CHARLS caregiver item, which was originally coded as 1 = caregiver present and 0 = no caregiver. For analytic interpretability, this item was reverse-coded so that caregiving status was coded as 0 = caregiver present and 1 = caregiver absent. Accordingly, higher values indicated caregiver absence. Because caregiving-related support is multidimensional (46, 47), this binary indicator should be understood as reflecting caregiving status only in the present analyses rather than a comprehensive measure of support adequacy, caregiver burden, or relationship quality (47, 48). Sex was coded from the original database record (0 = male, 1 = female). These variables were not included in the primary 19-node symptom-biomarker network but were introduced separately in subsequent analyses.

#### 2.2.4 Demographic and health characteristics

Additional variables included age, education, marital status, employment status, smoking, and alcohol use. These variables were not entered into the network as nodes but were used to describe the discovery and independent hospital cohorts and, where necessary, to support supplementary analyses.

### 2.3 Data preprocessing

Missing-data handling was determined according to each cohort’s analytic role and missing-data structure (32, 49). In the CHARLS discovery cohort, complete-case analysis was retained because the primary analysis aimed to estimate the empirical conditional association structure among all 19 symptom and biomarker nodes using a common set of observed variables. Because GGM estimation relies on the observed variance-covariance structure (50), imputing a relatively large block of biomarker nodes could introduce model-dependent covariance structures and affect edge weights or network topology. As missingness in CHARLS was concentrated mainly in the biomarker module, participants with complete data on all CES-D-10 items, the nine biomarkers, and the three external factors were retained. Variable-level missingness and comparisons between included and excluded CHARLS participants are reported in **Supplementary Table 3** and **S1**, respectively.

In the independent hospital cohort, multiple imputation was used because missingness was limited and concentrated in HbA1c (10 patients, 2.0%) and CysC (11 patients, 2.2%). Missing values were imputed using multiple imputation by chained equations with 20 imputed datasets, using a model that included all symptom nodes, biomarkers, external factors, and available demographic variables (32). Complete-case analyses were additionally performed in 485 patients with complete HbA1c and CysC data as a sensitivity check. TG and CRP were natural log-transformed; other continuous biomarkers were standardized directly, and all continuous biomarkers were converted to z scores. BMI values outside the clinically plausible range (<10 or >60 kg/m²) were treated as invalid. Transformed variable names are provided in **Supplementary Table 2**.

### 2.4 Network analysis

**Figure 1:**
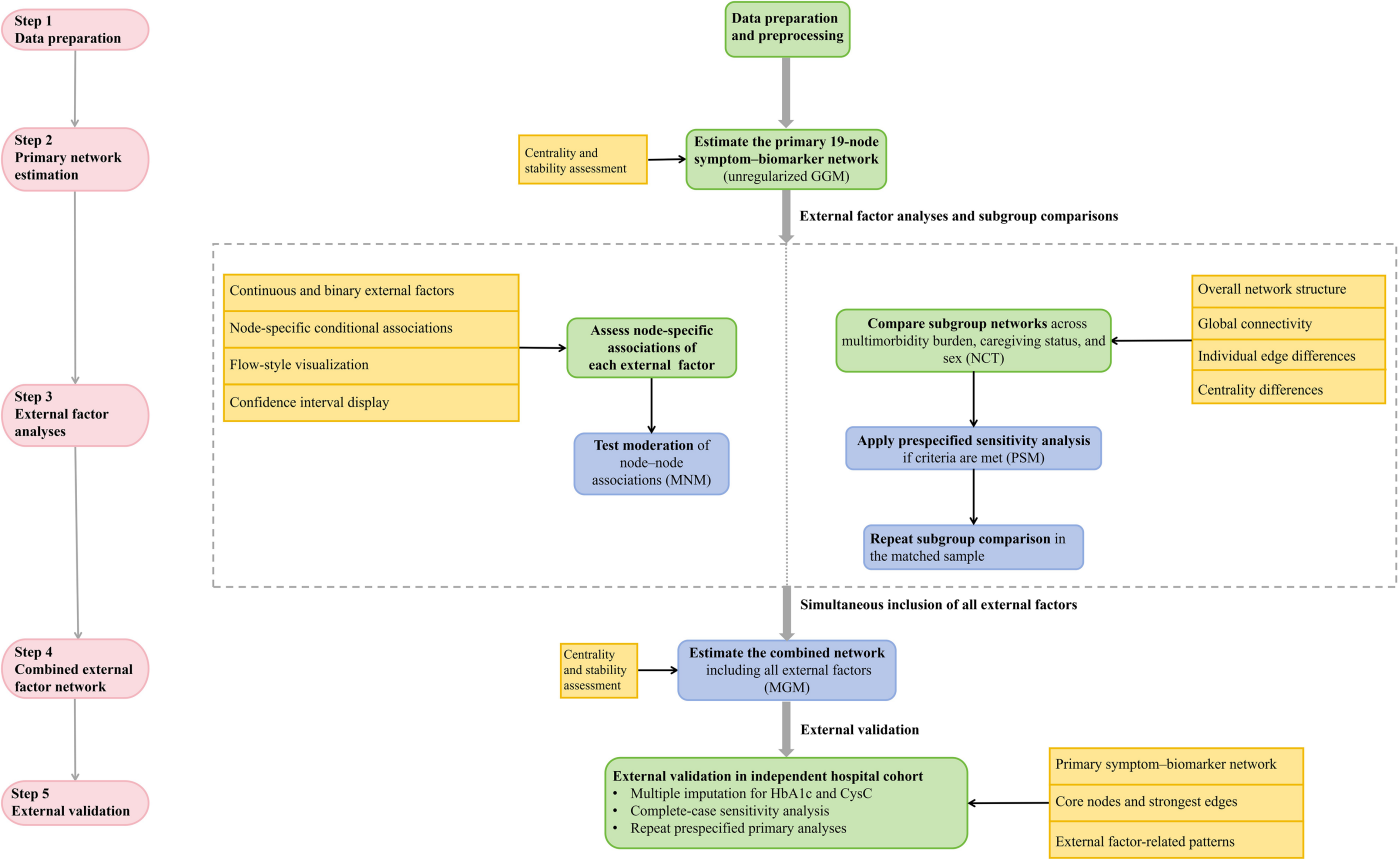
Simplified analytical workflow of the network analyses. The figure summarizes the main analytical steps at a conceptual level, including data preparation and preprocessing, estimation of the primary symptom-biomarker network, analyses of individual external factors, subgroup network comparison, estimation of the combined network including all external factors, and external validation in the independent hospital cohort. Detailed estimators, software packages, and technical specifications are described in the Methods section. GGM, Gaussian graphical model; MGM, mixed graphical model; MNM, moderated network model; NCT, Network Comparison Test; PSM, propensity score matching; HbA1c, glycated hemoglobin; CysC, cystatin C.

A series of network-analytic methods were used to examine associations involving the external factors. To improve readability, we first provide a simplified conceptual workflow of the analytical procedures (**Figure 1**), followed by detailed descriptions of each analytical step below.

#### 2.4.1 Estimating network

This step aimed to characterize the primary conditional association structure among depressive symptom and biomarker nodes before introducing the external factors. All exploratory networks were estimated in R using the bootnet package (version 1.6) (50). Networks including dichotomous variables, such as sex and caregiving status, were estimated using mixed graphical models (MGM; estimator = “mgm”) (51). Networks containing continuous or ordinal variables were estimated using Gaussian graphical models (GGM) (52). The primary 19-node symptom-biomarker network, comprising 10 ordinal CES-D items and continuous biomarker variables, was estimated using an unregularized GGM with estimator = “ggmModSelect” (53, 54).

For networks that included mixed variable types, MGM was used because it can estimate conditional associations among Gaussian, categorical, and binary variables within a single pairwise Markov random field (51). In this framework, continuous biomarker variables were treated as Gaussian nodes, CES-D items were treated as categorical/ordinal nodes, and binary variables such as sex and caregiving status were treated as two-level categorical nodes. Conditional associations are estimated using nodewise generalized regression models with an appropriate conditional distribution for each node. Therefore, an edge between a binary and a continuous node reflects their conditional interaction after adjustment for all other nodes in the network, rather than a conventional Pearson correlation.

Because the parameterization of MGM differs across edge types, MGM edge weights are not numerically equivalent to the partial correlation coefficients estimated in a GGM (51, 52). In the present study, GGM edge weights in the 19-node symptom-biomarker network were interpreted as partial correlations, whereas MGM edge weights involving binary or categorical nodes were interpreted as conditional interaction weights within the mixed graphical model. Accordingly, MGM-based edge weights were used to describe the presence, sign, and relative prominence of associations within the same MGM network, but were not directly compared in magnitude with GGM partial correlation coefficients across different network models.

#### 2.4.2 Conditional associations between external factors and network nodes

This step examined which specific symptom or biomarker nodes were conditionally associated with each external factor after accounting for the full symptom-biomarker network structure. These associations were evaluated using confirmatory network analysis implemented in psychonetrics (version 0.13.2) (55, 56). The confirmatory model was based on the exploratory network, with absent edges fixed at 0 and nonzero edges freely estimated. This approach quantified associations between each external factor and specific nodes while accounting for the full 19-node symptom-biomarker network structure (52). For the three external factors, associations with all 19 nodes were examined, resulting in 57 node-level tests. Statistical significance was evaluated using an overall Bonferroni-corrected threshold of p < 0.05/57 (p < 8.77 × 10^-4^).

#### 2.4.3 Network visualization

This step was used to display the estimated networks and external factor-node associations in a visually interpretable form. Networks were visualized using multidimensional scaling (MDS) in the networktools package (version 1.6.0), in which distances between nodes reflect association strength (57). Associations between each external factor and the 19 network nodes were further displayed as flow-style networks using qgraph (version 1.9.8) (58).

#### 2.4.4 Centrality and predictability measures

This step described the relative statistical connectedness of nodes within the estimated networks. Node centrality was calculated using qgraph, focusing on strength and expected influence (59). In the present study, these indices were used as descriptive measures of statistical connectedness within the estimated cross-sectional network rather than as indicators of causal influence, temporal precedence, or therapeutic priority. Strength was defined as the sum of absolute edge weights connected to a node, whereas expected influence retained edge signs and reflected the extent to which a node was positively or negatively connected to other nodes (59, 60). Closeness and betweenness were not examined because they may yield misleading interpretations in psychological networks (61). To complement centrality indices, and following previous recommendations for reporting node predictability in psychological network models (62, 63), node predictability was additionally estimated for each node in the 19-node symptom-biomarker network. Predictability was quantified as the nodewise R², representing the proportion of variance in a given node explained by its neighboring nodes in the estimated network. This index was used to describe how strongly each node was statistically accounted for by the surrounding network structure. As with centrality indices, predictability was interpreted descriptively and was not taken as evidence of causal controllability, temporal precedence, or intervention effects. Node predictability results are reported in **Supplementary Table 4**.

#### 2.4.5 Stability assessment

This step assessed whether the estimated edge weights and centrality indices were sufficiently stable to support descriptive interpretation. Network accuracy and stability were evaluated using ‘bootnet’ (version 1.6). Edge-weight accuracy was assessed using 95% confidence intervals based on 1,000 nonparametric bootstrap samples, and centrality stability was evaluated using a case-dropping subset bootstrap procedure and the correlation stability coefficient (50). Because centrality estimates can be sensitive to sampling variation, centrality findings were interpreted cautiously and as exploratory indicators of network position.

#### 2.4.6 Moderation analysis

This step tested whether the external factors statistically moderated symptom-symptom associations, beyond their direct conditional associations with individual symptom or biomarker nodes. After estimating direct conditional associations between the external factors and network nodes, moderated network models (MNM) were used to test whether the presence or strength of conditional associations among depressive symptoms varied by these factors. This analysis was conducted using the mgm package (version 1.2-15) (64).

#### 2.4.7 Network comparison tests and sensitivity analysis

This step evaluated whether networks differed between subgroups defined separately by each external factor, including differences in overall network structure, global connectivity, individual edges, and node strength. The comparison subgroups were defined by the external factors. multi-morbidity burden was categorized as low (0–1 conditions other than heart disease) or high (≥4), with the intermediate group (2–3) excluded. Sex and caregiving status were analyzed as binary variables. In the original dataset, caregiver presence was coded as 1 and absence as 0; for the present analyses, this variable was reverse-coded such that caregiving status was coded as 0 = caregiver present and 1 = caregiver absent. To reduce subgroup-size imbalance, random downsampling was applied for sex and caregiving status (seed = 123; sex: n = 679/group; caregiving status: n = 449/group), whereas multi-morbidity comparisons retained the original subgroup sizes (low: n = 439; high: n = 497) (65). Original and final subgroup sizes are shown in **Supplementary Table 5**.

Subgroup networks were compared using the Network Comparison Test (NCT; version 2.2.2) (66) with 2,000 permutations. We examined differences in network structure, global strength, edge weights, and node strength, with edge-level p values adjusted by the false discovery rate (67). Because depressive symptom burden may be associated with network characteristics (68, 69), propensity score matching (PSM) was prespecified as a sensitivity analysis to reduce potential confounding by depressive symptom burden and was applied only when both the total CES-D difference and the initial NCT result for network structure were significant. NCT was then repeated in the matched sample using MatchIt (version 4.7.2) (70).

#### 2.4.8 Combined network including all external factors

This step examined the relative positioning of the three external factors within a single network model. A combined external-factor network was estimated by including the 19 depressive symptom and biomarker nodes together with multi-morbidity burden, caregiving status, and sex. Because this network included binary variables, it was estimated using an MGM framework. Given the mixed parameterization of MGM edge weights, edge weights and node centrality in the combined external-factor network were interpreted descriptively within that network and were not directly compared in magnitude with GGM partial correlations from the primary 19-node network. Node centrality and stability were assessed descriptively using the same principles described above.

#### 2.4.9 External replication in the independent hospital cohort

This final step assessed whether the major network patterns observed in the discovery cohort were reproducible in an independent clinical sample. Key analyses were repeated in the independent hospital cohort using the same variable definitions, coding rules, preprocessing procedures, and network estimation strategy as far as possible (71). This step assessed external replicability of the primary network structure, core nodes, strongest edges, and external factor-related patterns based on consistency in direction and relative prominence across cohorts (21). Analyses were conducted in R 4.5.2, and code is available from the corresponding author upon reasonable request.

## 3 Results

### 3.1 Sample characteristics of the discovery and independent hospital cohorts

The CHARLS discovery cohort comprised 1,685 participants, and the independent hospital cohort comprised 506 patients. The median ages of the two cohorts were 63.0 [56.0, 69.0] and 63.0 [56.0, 70.0] years, respectively. Women accounted for 59.7% and 60.3% of the two cohorts, respectively. Median CES-D-10 total scores were 8.0 [4.0, 14.0] and 10.0 [5.0, 16.0], and the proportions with CES-D-10 scores ≥10 were 41.8% and 52.0%, respectively. Additional descriptive characteristics are presented in **Supplementary Table 6**.

### 3.2 Discovery sample: 19-node symptom-biomarker network

**Figure 2:**
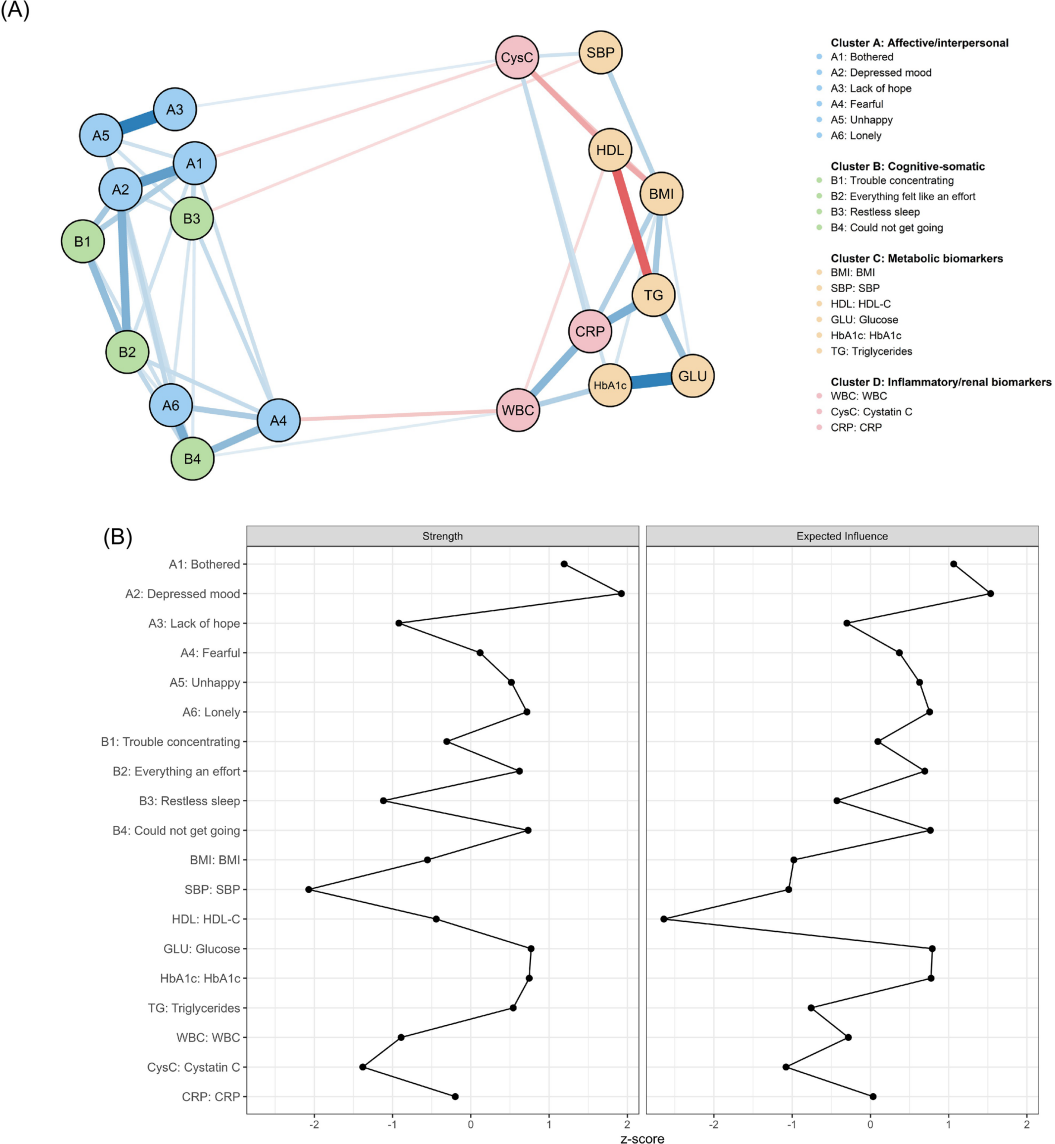
Nineteen-node symptom-biomarker network in the CHARLS discovery cohort. **(A)** Network of depressive symptoms and biomarkers in the CHARLS discovery cohort. Depicted in the MDS graph layout, where the proximity between nodes was proportional to edge strength. Blue edges indicate positive relationships; red edges indicate negative relationships. Light blue nodes denote affective/interpersonal depressive symptoms; light green nodes denote cognitive-somatic depressive symptoms; tan nodes denote metabolic biomarkers; pink nodes denote inflammatory/renal biomarkers. **(B)** Node centrality. The x-axis represents the z-score. Depressive symptoms: A1, Bothered by small things; A2, Depressed mood; A3, Lack of hope about the future; A4, Feeling fearful; A5, Unhappy; A6, Lonely; B1, Trouble concentrating; B2, Everything felt like an effort; B3, Restless sleep; B4, Could not get going. Biomarkers: BMI, body mass index; SBP, mean systolic blood pressure; WBC, white blood cell count; HDL, high-density lipoprotein cholesterol; GLU, fasting glucose; CysC, cystatin C; HbA1c, glycated hemoglobin; TG, triglycerides; CRP, C-reactive protein.

We first estimated the 19-node symptom-biomarker network (**Figure 2A**). The network showed clear within-domain clustering and relatively sparse cross-domain connections (edge density = 0.29). The strongest symptom edges were between unhappy (A5) and lack of hope about the future (A3), and between depressed mood (A2) and bothered by small things (A1), whereas the strongest biomarker edges were between HbA1c and GLU, TG and HDL-C, and CRP and WBC. Symptom nodes clustered into affective/interpersonal (A1-A6) and cognitive/somatic (B1-B4) groups, and biomarker nodes clustered into metabolic-related and inflammatory/renal-function-related groups. Depressed mood (A2) showed the highest centrality among symptom nodes, followed by bothered by small things (A1), could not get going (B4), and lonely (A6) (**Figure 2B**), while GLU and HbA1c showed relatively high centrality among biomarkers (**Supplementary Table 7**). Node predictability showed a broadly consistent pattern. In the discovery cohort, depressed mood (A2) had the highest predictability (R² = 0.491), followed by bothered by small things (A1; R² = 0.413), everything felt like an effort (B2; R² = 0.393), could not get going (B4; R² = 0.371), and lonely (A6; R² = 0.352). These results indicate that the most predictable nodes were mainly depressive symptom nodes, particularly affective and cognitive-somatic symptoms embedded in the symptom subnetwork (**Supplementary Table 4**).

### 3.3 Discovery sample: individual external factors in the symptom-biomarker network

#### 3.3.1 Multi-morbidity burden

**Figure 3:**
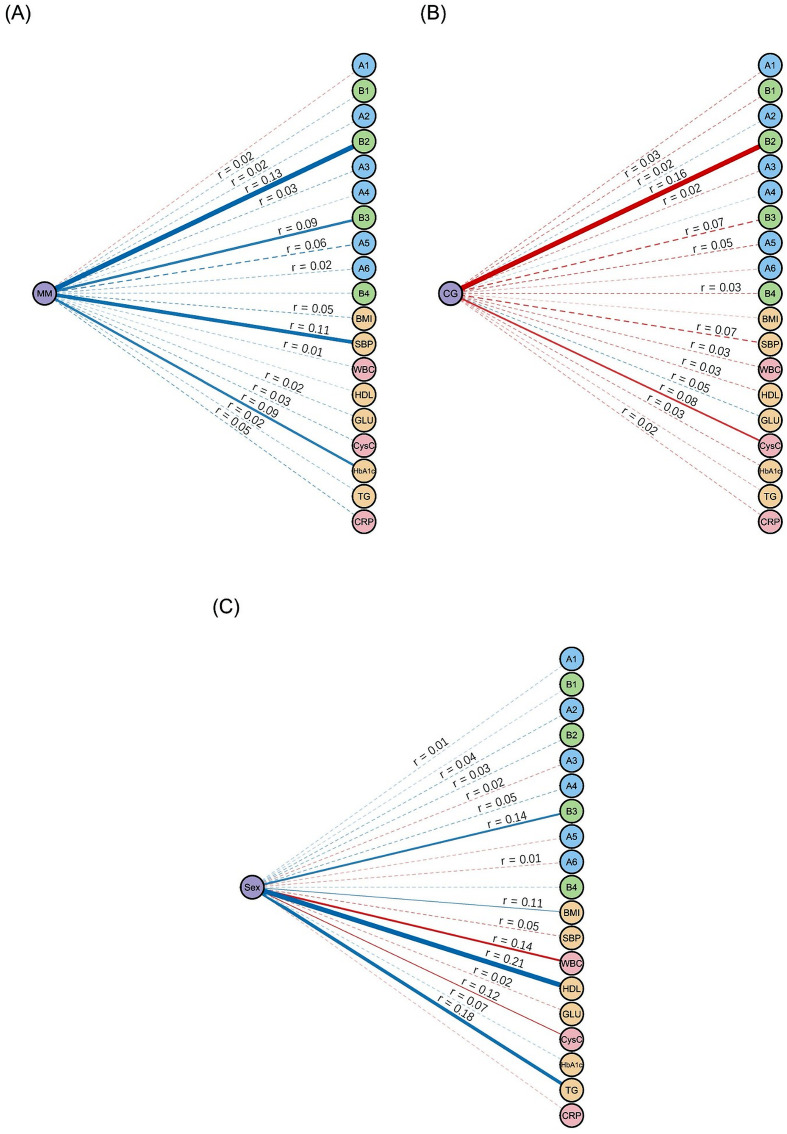
Individual external factors in the 19-node symptom-biomarker network in the CHARLS discovery cohort. Conditional associations are shown between network nodes and **(A)** multimorbidity burden (MM), **(B)** caregiving status (CG), and **(C)** sex. Purple nodes denote external factors; light blue nodes denote affective/interpersonal depressive symptoms; light green nodes denote cognitive-somatic depressive symptoms; tan nodes denote metabolic biomarkers; pink nodes denote inflammatory/renal biomarkers. Blue edges indicate positive associations, and red edges indicate negative associations. Solid lines denote edges significant after overall Bonferroni correction across all external factor-node tests (57 tests, p < 8.77 × 10^-4^); dashed lines denote edges that did not pass this threshold. Thicker lines indicate stronger associations. Depressive symptoms: A1, bothered by small things; A2, depressed mood; A3, lack of hope about the future; A4, feeling fearful; A5, unhappy; A6, lonely; B1, trouble concentrating; B2, everything felt like an effort; B3, restless sleep; B4, could not get going. Biomarkers: BMI, body mass index; SBP, mean systolic blood pressure; WBC, white blood cell count; HDL, high-density lipoprotein cholesterol; GLU, fasting glucose; CysC, cystatin C; HbA1c, glycated hemoglobin; TG, triglycerides; CRP, C-reactive protein.

Multi-morbidity burden was associated with a broad cross-domain association pattern (**Figure 3A**), with the strongest positive associations involving everything felt like an effort (B2), restless sleep (B3), SBP, and HbA1c. All four remained significant after multiple-testing correction.

#### 3.3.2 Caregiving status

Caregiving status was associated with a localized association pattern (**Figure 3B**), with the strongest negative associations involving everything felt like an effort (B2) and CysC. Both remained significant after multiple-testing correction.

#### 3.3.3 Sex

Sex was associated with a biomarker-oriented association pattern (**Figure 3C**). Its strongest positive associations were with restless sleep (B3), HDL-C, and TG, and its strongest negative associations were with WBC and CysC. After multiple-testing correction, associations with restless sleep (B3), BMI, WBC, HDL-C, CysC, and TG remained significant. Detailed node-level estimates, raw p values, Bonferroni-adjusted p values, and significance status for all 57 external factor-node tests in the CHARLS discovery cohort are presented in **Supplementary Table 8**.

#### 3.3.4 Comparative summary of individual external factor effects

Taken together, the three external factors were associated with distinct association profiles. multi-morbidity burden was associated with the broadest cross-domain pattern, caregiving status was associated with more localized associations, and sex was linked mainly to biomarker nodes. No significant moderation effects or between-group differences in network structure or global connectivity were observed; accordingly, the prespecified PSM sensitivity analysis was not performed (**Supplementary Table 9, S10**).

### 3.4 Discovery sample: combined network including all three external factors

**Figure 4:**
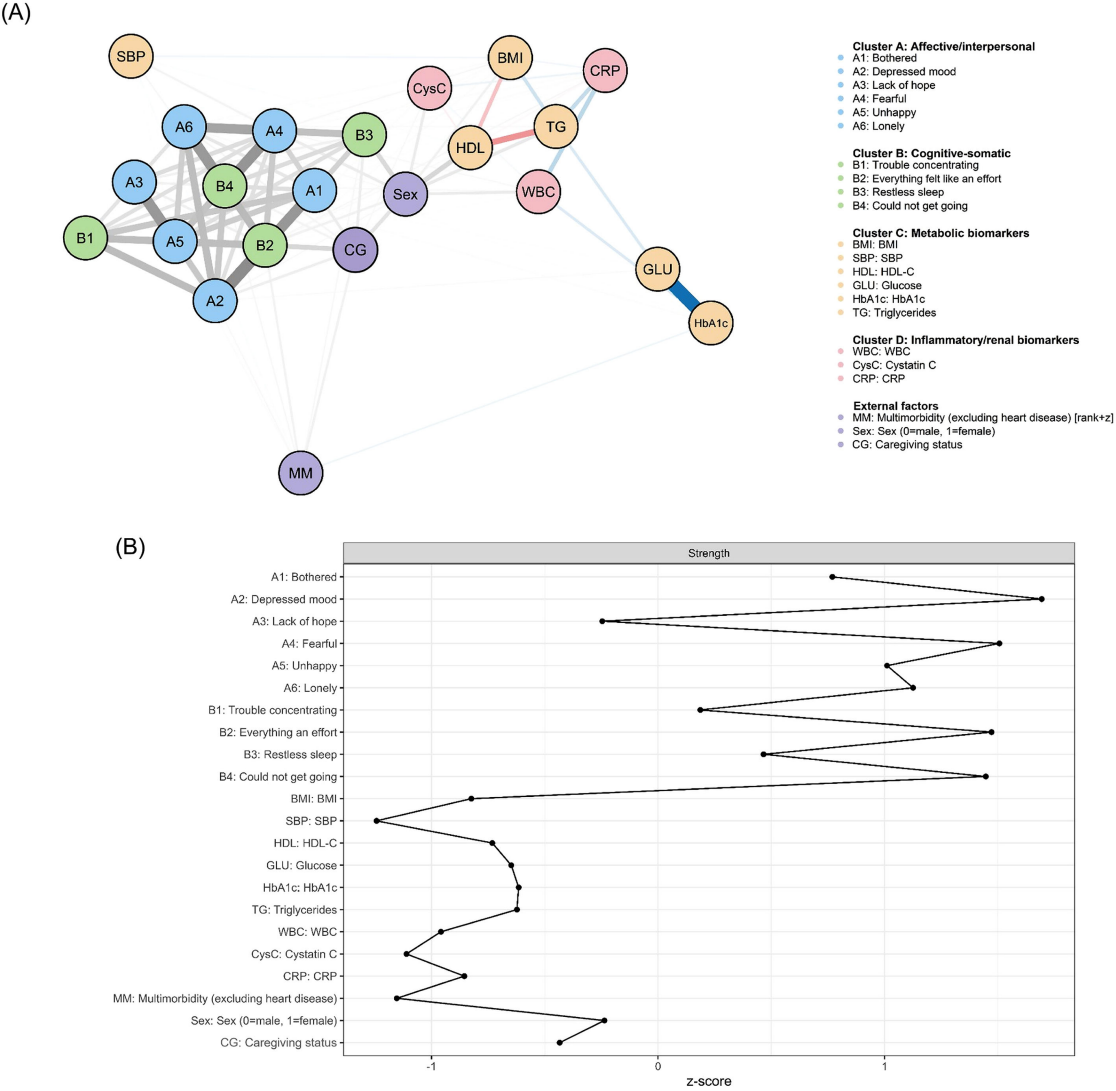
Combined network including depressive symptom-biomarker nodes and external factors in the CHARLS discovery cohort. **(A)** Full mixed graphical model (MGM) network including 10 CES-D-10 symptom nodes, 9 biomarker nodes, and three external factors: multimorbidity burden (MM), caregiving status (CG), and sex. Blue edges indicate positive conditional associations, red edges indicate negative conditional associations, and thicker edges indicate larger absolute edge weights within the MGM. Node colors denote affective/interpersonal symptoms, cognitive-somatic symptoms, metabolic biomarkers, inflammatory/renal biomarkers, and external factors. **(B)** Node strength in the full MGM network. Because this network included binary/categorical and Gaussian nodes, MGM edge weights represent conditional interaction weights and should not be interpreted as GGM partial correlation coefficients or directly compared in magnitude with GGM edge weights from the primary 19-node network. MM, multimorbidity burden excluding heart disease; CG, caregiving status; coded as 0 = caregiver present and 1 = caregiver absent; sex = 0 male and 1 female; CES-D-10, Center for Epidemiologic Studies Depression Scale, 10-item version; GGM, Gaussian graphical model; MGM, mixed graphical model.

In the combined network, sex was positioned closest to the network center and was connected to restless sleep (B3), BMI, HDL-C, TG, WBC, CysC, and caregiving status (CG) (**Figure 4A**). Caregiving status was connected to everything felt like an effort (B2), CysC, and sex, whereas multi-morbidity burden was linked to everything felt like an effort (B2), restless sleep (B3), SBP, and HbA1c. Within the full MGM network, depressed mood (A2) showed the highest centrality, and sex showed the highest strength among the three external factors (**Figure 4B**). Signed expected influence is reported in **Supplementary Table 11**.

### 3.5 External replication of major patterns in the independent hospital cohort

#### 3.5.1 Independent hospital cohort: 19-node symptom-biomarker network

In the independent hospital cohort, the 19-node symptom-biomarker network showed a broadly similar organization to that in the discovery cohort (**Supplementary Table 12**), with closely interconnected symptom nodes, compact biomarker clusters, and relatively sparse cross-domain connections. Several of the strongest within-domain edges were reproduced, including unhappy (A5) and lack of hope about the future (A3), bothered by small things (A1) and depressed mood (A2), TG and HDL-C, HbA1c and GLU, and CRP and WBC. Depressed mood (A2) again showed the highest centrality among symptom nodes and was the only symptom that consistently ranked first across cohorts. TG, HDL-C, and CRP also showed relatively high centrality among biomarkers. Complete-case sensitivity analyses in the validation cohort yielded broadly similar centrality patterns for the main replicated nodes, particularly for depressed mood, which remained the highest-ranking symptom node (**Supplementary Table 12**). Node predictability in the independent hospital cohort further supported the cross-cohort prominence of depressed mood. Depressed mood (A2) showed the highest predictability in both the multiple-imputation analysis (R² = 0.468) and the complete-case sensitivity analysis (R² = 0.468). Triglycerides and HDL-C also showed relatively high predictability in the validation cohort, consistent with the stronger lipid-related biomarker clustering observed in this sample (**Supplementary Table 4**).

#### 3.5.2 Independent hospital cohort: external factors in the symptom-biomarker network

**Figure 5:**
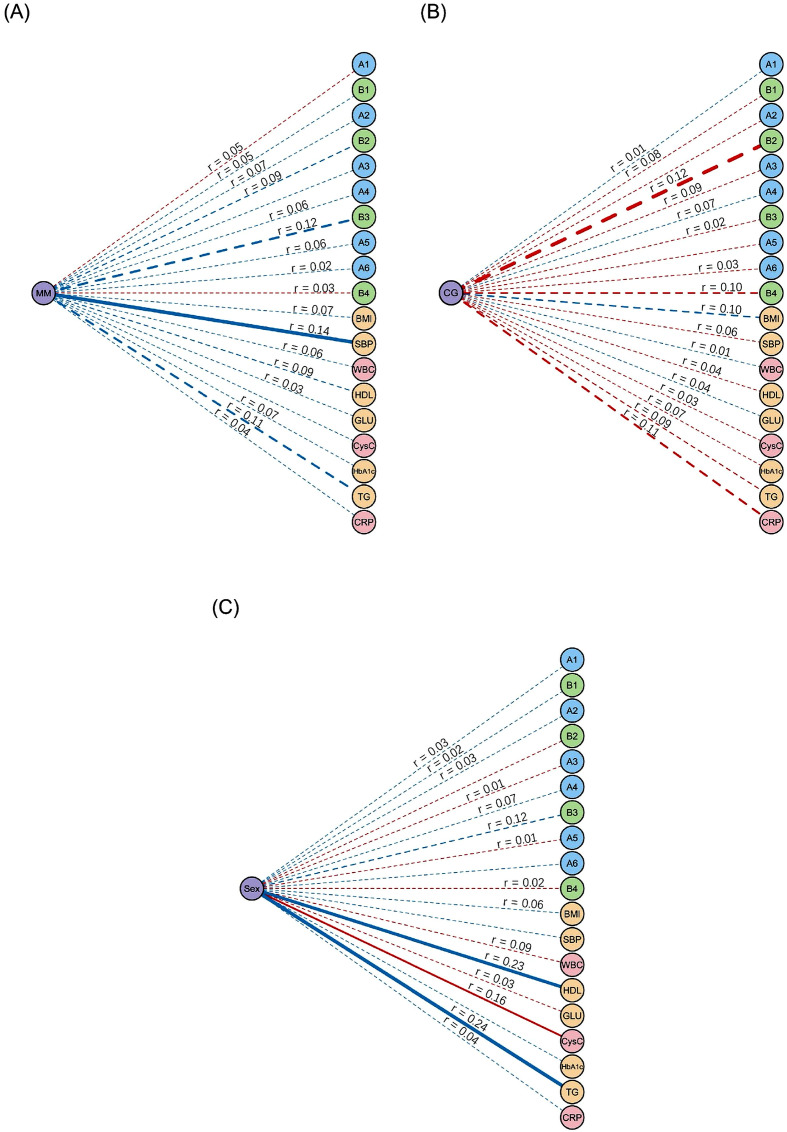
Individual external factors in the 19-node symptom-biomarker network in the independent hospital cohort. Conditional associations are shown between network nodes and **(A)** multimorbidity burden (MM), **(B)** caregiving status (CG), and **(C)** sex. Purple nodes denote external factors; light blue nodes denote affective/interpersonal depressive symptoms; light green nodes denote cognitive-somatic depressive symptoms; tan nodes denote metabolic biomarkers; pink nodes denote inflammatory/renal biomarkers. Blue edges indicate positive associations, and red edges indicate negative associations. Solid lines denote edges significant after overall Bonferroni correction across all external factor-node tests (57 tests, p < 8.77 × 10^-4^); dashed lines denote edges that did not pass this threshold. Thicker lines indicate stronger associations. Depressive symptoms: A1, bothered by small things; A2, depressed mood; A3, lack of hope about the future; A4, feeling fearful; A5, unhappy; A6, lonely; B1, trouble concentrating; B2, everything felt like an effort; B3, restless sleep; B4, could not get going. Biomarkers: BMI, body mass index; SBP, mean systolic blood pressure; WBC, white blood cell count; HDL, high-density lipoprotein cholesterol; GLU, fasting glucose; CysC, cystatin C; HbA1c, glycated hemoglobin; TG, triglycerides; CRP, C-reactive protein.

The independent hospital cohort provided differential support for the external factor associations observed in the discovery cohort (**Figures 5A–C**). For multi-morbidity burden, the strongest associations again involved everything felt like an effort (B2), restless sleep (B3), and SBP, although only SBP remained significant after multiple-testing correction (**Figure 5A**). For caregiving status, associations were weaker and none remained significant after correction (**Figure 5B**). By contrast, associations involving sex formed the clearest and most stable cross-cohort pattern, concentrated mainly in the biomarker domain: positive associations with HDL-C and TG and a negative association with CysC remained significant after multiple-testing correction, whereas restless sleep (B3) showed only a weaker symptom-level signal (**Figure 5C**). Overall, replication was strongest for sex, partial for multi-morbidity burden, and limited for caregiving status. Detailed node-level estimates, raw p values, Bonferroni-adjusted p values, and significance status for all 57 external factor-node tests in the independent hospital cohort are presented in **Supplementary Table 13**.

## 4 Discussion

The present study indicates that, in middle-aged and older adults with heart disease, depressive heterogeneity was reflected more clearly in reproducible local associations involving external factors within a joint depressive symptom-biomarker network than in broad network-wide differences. Because the analyses were cross-sectional, these associations should be interpreted as conditional statistical associations and do not imply that multi-morbidity burden, caregiving status, or sex temporally preceded or causally influenced depressive symptoms or biomarker alterations. Across cohorts, multi-morbidity burden showed the broadest cross-domain pattern, caregiving status showed weaker and less stable associations, and sex showed the clearest biomarker-oriented pattern. These findings extend previous work focused on symptoms alone or single biomarkers by suggesting that integrating depressive symptoms, biomarkers, and clinically relevant external factors within a joint network framework may provide a more clinically interpretable characterization of depressive phenotypes in this population.

### 4.1 Multi-morbidity burden

Among the three external factors, multi-morbidity burden showed the broadest association pattern, concentrated mainly on everything felt like an effort, restless sleep, and cardiometabolic indicators such as SBP and HbA1c. In the independent hospital cohort, the directions of association for everything felt like an effort, restless sleep, and SBP were generally consistent, with TG also showing a prominent role, suggesting partial cross-cohort stability. These findings indicate that, in middle-aged and older adults with heart disease, multi-morbidity burden was reflected less in a generalized increase in depressive symptom burden than in selective cross-domain associations involving reduced drive, sleep problems, and cardiometabolic abnormalities.

This pattern is consistent with evidence that multi-morbidity reflects not only the number of coexisting diseases but also greater illness burden, treatment demands, and functional impairment (44, 72). We did not observe broad reorganization of the overall network structure in relation to multi-morbidity burden, suggesting that its associations may be expressed mainly as local connections with specific symptom-biomarker nodes. This interpretation is also plausible because multi-morbidity is itself heterogeneous, and different combinations of chronic conditions may not have the same associations with depressive symptom profiles (73, 74). Clinically, middle-aged and older adults with heart disease and high multi-morbidity burden should not be evaluated solely on the basis of total depression scores or within a single-disease framework. Greater attention may be warranted for individuals who also present with reduced energy or motivation, restless sleep, and abnormalities in blood pressure or glucose/lipid metabolism (75).

### 4.2 Caregiving status

Compared with multi-morbidity burden, caregiving status was associated with weaker, more localized patterns. In the discovery cohort, the main associations involved everything felt like an effort and CysC; in the independent hospital cohort, no edges remained significant after Bonferroni correction, although everything felt like an effort and several other nodes showed trend-level associations. This pattern suggests that caregiving status was more likely to appear as limited and unstable local connections than as a stable network-wide pattern.

These findings should be interpreted cautiously. In the present study, caregiving status was derived from a binary indicator of caregiver presence versus absence. However, caregiving-related support is multidimensional and may involve structural availability, functional and emotional support, relationship quality, and caregiver burden (46, 47). Recent literature also suggests that caregiving burden and social support are related but non-equivalent constructs. Higher caregiving burden is associated with poorer psychological well-being, whereas greater social support appears protective; similarly, among family caregivers of home-dwelling older adults with disability, social support and positive caregiving experience predict lower depressed mood, whereas caregiving burden predicts higher depressed mood (76, 77). However, direct analyses of how these caregiving dimensions are embedded in joint depressive symptom-biomarker networks in older adults with heart disease remain limited. Taken together, these findings support interpreting caregiver presence versus absence as a structural caregiving-context indicator rather than a direct proxy for support adequacy or caregiving burden. In heart failure settings, relationship quality has also been linked to caregiver benefit finding and caregiver burden (48). Accordingly, the observed associations in the present study may reflect not only support availability but also care needs, functional status, or broader illness burden. This may help interpret the negative association with everything felt like an effort in both samples, because caregiver presence may mark greater dependency or poorer physical functioning rather than a protective social resource per se. No significant differences in overall network structure or global connectivity were observed, suggesting that caregiving status may be better understood as a background contextual indicator than as a stable network-wide pattern (73). Clinically, caregiving status may need to be considered together with reduced energy, activity limitation, sleep disturbance, and related physiological abnormalities when assessing long-term self-management needs (78, 79).

### 4.3 Sex

Among the three external factors, associations involving sex formed the clearest and most stable pattern, concentrated mainly in the biomarker domain. In the discovery cohort, sex was associated with HDL-C, TG, WBC, CysC, and BMI, with only limited symptom-level associations, among which restless sleep was the most prominent; in the independent hospital cohort, associations with HDL-C, TG, and CysC remained significant after multiple-testing correction, whereas the symptom-level signal for restless sleep was weaker. Taken together, these findings indicate that, in middle-aged and older adults with heart disease, sex-related differences were reflected more in selective embedding within metabolic- and renal-function-related pathways than in broad symptom-level associations.

This pattern has biological plausibility. HDL-C and TG may be sensitive markers of sex-related cardiovascular risk, whereas the continued involvement of CysC is consistent with its role as an indicator of renal function and cardiorenal risk (80–82). By contrast, restless sleep was one of the few symptom nodes that remained related to sex, suggesting that sleep-related symptoms may represent one of the limited symptom domains in which sex-related differences were observed (83–85). Clinically, sex may be better understood as the clearest biomarker-oriented contextual factor in this joint network, rather than as a marker of broad symptom-level differences or reorganization of the overall network structure.

### 4.4 Comparison of associations involving different external factors

Multi-morbidity burden, caregiving status, and sex were associated with distinct conditional patterns rather than a single pattern broadly related to depressive symptom burden. Associations involving multi-morbidity burden were the broadest across domains, associations involving caregiving status were weaker and more localized, and associations involving sex formed the clearest and most stable biomarker-oriented pattern. No significant differences in overall network structure or global connectivity were observed, suggesting that these associations were expressed more at the node level than as broad network reorganization. Because this was a group-level conditional association network, the findings should not be directly generalized to individual-level dynamic processes (86).

These findings should also be interpreted in the context of recent network-analysis and prediction-modeling studies in related aging and cardiometabolic populations. A recent nationwide population-based network analysis of older adults with multi-morbidity examined depressive symptoms together with cognitive performance and identified central and bridge symptoms, supporting the value of symptom-level network analysis in multimorbid aging populations rather than relying only on total depression scores (23). In cardiovascular and metabolic populations, recent machine-learning work has developed validated depression-detection models and identified sleep disturbance, life satisfaction, and loneliness as important predictors, indicating that depression in these patients is embedded in multidimensional clinical, behavioral, and psychosocial contexts (24). Similarly, a recent deep-learning study using CHARLS with independent validation in CLHLS highlights the growing emphasis on externally validated prediction frameworks in geriatric depression research (87). Although the present study was not designed as a machine-learning prediction model, it complements this prediction-oriented literature by using a network framework to examine group-level conditional associations among depressive symptoms, routinely available biomarkers, and external factors. The broader cardiovascular relevance of this perspective is supported by recent evidence linking frailty and depressive symptoms with cardiovascular disease risk in middle-aged and older adults (15). Most directly, a multilayer network analysis of cardiovascular-depression comorbidity identified symptom-specific molecular biomarkers, reinforcing the view that biological correlates of depression may differ across individual symptoms rather than mapping uniformly onto total depressive burden (21). Compared with these studies, the present work extends the literature by integrating depressive symptom nodes, clinical biomarkers, and external factors within a joint symptom-biomarker network and by examining whether the main local patterns were reproducible in an independent hospital cohort.

### 4.5 Centrality patterns in the symptom-biomarker network

Across networks, depressed mood showed the highest centrality among symptom nodes and was the only symptom that consistently ranked first across cohorts. This finding indicates that depressed mood occupied a relatively prominent position within the estimated cross-sectional group-level networks. However, this result should be interpreted cautiously. Higher centrality does not establish that depressed mood is a causal driver of other symptoms or biomarkers, nor does it imply that it is necessarily the most clinically important symptom or the optimal intervention target. Rather, the repeated prominence of depressed mood across cohorts suggests that negative affect was a reproducible feature of the estimated network architecture in this population. Accordingly, the centrality findings should be understood as exploratory and hypothesis-generating, and future longitudinal and interventional studies are needed to determine whether nodes with higher centrality also have causal or therapeutic relevance. The added predictability analysis provided complementary information by showing how strongly each node was statistically explained by its neighboring nodes. Depressed mood also showed the highest predictability in both cohorts, whereas lipid-related biomarkers showed relatively high predictability in the validation cohort. Clinically relevant nodes such as restless sleep and inflammatory biomarkers can also be evaluated in this framework because their predictability indicates the extent to which they are statistically embedded in the observed network. However, high predictability should not be interpreted as evidence that intervening on sleep, inflammation, or any other node would causally change neighboring nodes or produce global network improvement. Rather, predictability should be viewed as an assessment-oriented and hypothesis-generating index that may help identify nodes more strongly embedded in the observed symptom-biomarker network (**Supplementary Table 4**).

### 4.6 Heart disease heterogeneity and network interpretation

The broad clinical definition of heart disease should be considered when interpreting the joint depressive symptom-biomarker network. In CHARLS, heart disease was identified by self-reported physician diagnosis rather than detailed cardiovascular phenotyping. This category likely includes heterogeneous cardiovascular conditions, including ischemic, failure-related, rhythm-related, and valvular disorders, which differ in symptom burden, inflammatory and metabolic activity, cardiac function, functional limitation, treatment exposure, and symptom perception (83, 88, 89). Importantly, these differences are not only diagnostic but also mechanistic. In heart failure, depression commonly co-occurs with fatigue, sleep disturbance, reduced activity, and functional limitation; in atrial fibrillation, psychological distress and altered symptom perception are particularly salient; and in older adults with aortic stenosis, depression is also clinically relevant and may follow a distinct course (90–92). In coronary disease, psychophysiological pathways linking depression and cardiac prognosis also appear to be symptom-sensitive rather than fully captured by total depression burden alone (93). Together, these findings suggest that the same symptom or biomarker node does not necessarily carry the same clinical meaning across all forms of heart disease. At the same time, direct phenotype-specific analyses of joint depressive symptom-biomarker networks remain limited. Current evidence therefore supports the clinical relevance of cardiovascular heterogeneity, but not yet a fully differentiated network map across specific cardiac phenotypes.

Such heterogeneity may be relevant to the estimated joint network in several ways. First, biomarker profiles can vary across cardiovascular subtypes and severity levels, and such variation may be associated with differences in the strength and direction of biomarker-symptom edges. Second, symptom profiles may also differ according to dyspnea, fatigue, sleep disturbance, functional limitation, treatment burden, and symptom perception, which may be accompanied by differences in symptom-symptom and symptom-biomarker associations (89–91, 94). Third, when clinically distinct cardiovascular conditions are combined into a single analytic group, subtype-specific associations can be averaged, weaker cross-domain edges can be attenuated, and the clinical specificity of pooled estimates can be reduced (50, 95, 96). This issue is particularly relevant in the present study because the network showed relatively sparse cross-domain connections and selective local associations rather than broad network-wide differences. In such a context, pooled estimates are especially sensitive to subtype-related variation in both biomarker profiles and symptom burden. Accordingly, nodes related to effort, sleep, inflammation, metabolism, and cardiorenal vulnerability should not be assumed to carry identical clinical meaning across all forms of heart disease, even when they occupy similar positions in the pooled network. This interpretation is also consistent with recent multilayer cardiovascular-depression network work showing that biomarker links can be symptom-specific rather than uniformly distributed across depression as a whole (21). Therefore, the present findings are best interpreted as population-level patterns among middle-aged and older adults with broadly defined heart disease, rather than as mechanisms specific to any single cardiovascular diagnosis. Future studies with more detailed cardiovascular phenotyping should compare pooled and subtype-specific networks, incorporate cardiac function and treatment-related characteristics, and examine whether depressive symptom-biomarker associations differ across cardiovascular subtypes, disease severity levels, and longitudinal stages of illness.

### 4.7 Clinical applicability and implementation considerations

Within these interpretive boundaries, the present findings have several implications for clinical assessment and care planning in cardiology, geriatric psychiatry, and multidisciplinary care settings (83). These implications should not be interpreted as a validated clinical decision tool, but as assessment-oriented considerations that require testing in longitudinal and interventional studies. First, the findings support symptom-focused assessment beyond total depression scores (21). Rather than identifying any single central symptom as a treatment target (60, 61), the network results highlight the value of distinguishing affective, motivational, somatic, and sleep-related presentations. In middle-aged and older adults with heart disease, symptoms such as everything felt like an effort and restless sleep are clinically relevant because they showed reproducible or interpretable associations with multimorbidity burden and cardiometabolic indicators, whereas depressed mood showed a consistently central statistical position across cohorts. This symptom-level approach can help clinicians avoid treating depressive burden as a uniform construct and instead identify patients whose depressive presentation is more closely embedded in fatigue-like, motivational, sleep-related, or affective disturbance.

Second, the factor-specific association patterns provide a framework for more structured clinical assessment. In patients with high multimorbidity burden, assessment should include reduced energy, sleep disturbance, blood pressure, and glycemic or lipid abnormalities, because multimorbidity burden was associated with the broadest cross-domain pattern (72, 73, 75). For caregiving status, the findings indicate that caregiver presence or absence should be interpreted together with functional limitation, care needs, and self-management capacity, rather than as a simple marker of support adequacy (47, 78, 79). For sex-related differences, the strongest and most reproducible associations were concentrated in biomarker nodes, particularly HDL-C, TG, and CysC, indicating that sex may be more informative as a biomarker-oriented contextual factor than as a broad marker of depressive symptom-level differences (80–82).

Third, these findings support a multidisciplinary assessment pathway. In cardiology or geriatric psychiatry settings, symptom-level depression screening can be combined with routinely available biomarkers and contextual information on multimorbidity and caregiving status (1, 83). Such an approach may help identify patients who require coordinated attention to depressive symptoms, cardiometabolic abnormalities, sleep problems, functional limitation, and long-term self-management needs. However, these applications remain preliminary. Because the present study was cross-sectional and based on group-level conditional associations, future longitudinal and interventional studies are needed before these network patterns can be interpreted as temporal mechanisms, intervention targets, or tools for individualized clinical decision-making (24, 71, 86).

### 4.8 Limitations

Several limitations should be noted. First, the clinical characterization of heart disease was limited, especially in the discovery cohort. In CHARLS, heart disease was identified using a self-reported physician-diagnosed chronic disease item, and detailed information on cardiovascular subtype, cardiac function, ejection fraction, New York Heart Association functional class, disease severity, and treatment characteristics was unavailable. This precluded subgroup analyses by cardiovascular diagnosis and adjustment for cardiac severity indicators. Accordingly, the estimated network should be interpreted as an aggregate pattern across heterogeneous cardiovascular conditions, and subtype-specific symptom-biomarker associations may have been obscured.

Second, the cross-sectional design precludes temporal or causal interpretation. The observed associations, including those involving external factors, should therefore be understood as conditional statistical associations rather than temporal or causal relationships. This caution also applies to centrality and predictability: nodes with higher values reflect greater statistical connectedness or local embedding within the estimated network, but do not necessarily represent causal drivers, clinically dominant symptoms, optimal intervention targets, or individualized clinical indicators. The clinical utility of the proposed symptom-focused and biomarker-informed assessment approach was also not directly tested; therefore, the suggested implications for clinical stratification, multidisciplinary assessment, and personalized care should be regarded as preliminary and hypothesis-generating.

Third, the discovery and independent hospital cohorts differed in sampling source and clinical context, so cross-cohort comparisons should be interpreted as external replication of major patterns rather than strict validation of every parameter. Fourth, caregiving status was measured using a binary indicator of caregiver presence versus absence. This measure did not capture caregiving source, frequency, quality, emotional adequacy, stability, relationship quality, perceived burden, or care needs (46, 47). Associations involving this variable should therefore not be interpreted as support deficiency alone, because they may also reflect care needs, functional limitation, or broader illness burden.

Fifth, only nine biomarkers were included and all were measured at a single time point, limiting coverage of broader physiological systems and dynamic biological change. Sixth, the CHARLS discovery network was estimated from complete cases. Although complete-case analysis was retained to avoid model-dependent imputation of a relatively large biomarker block, the resulting network represents the complete-case analytic sample rather than all eligible participants with heart disease. Seventh, the independent hospital cohort was modest in size for a 19-node symptom-biomarker network with external factor analyses and Network Comparison Tests. Limited statistical power may have reduced the ability to detect weak edges, small node-level associations, and subtle between-network differences; nonsignificant validation findings should therefore be interpreted cautiously. Finally, unmeasured factors such as medication use, lifestyle, and treatment-related characteristics may also have been associated with the estimated network patterns. Future studies should use larger samples, broader physiological and social support measures, detailed cardiovascular phenotyping, and longitudinal designs.

## 5 Conclusion

In middle-aged and older adults with heart disease, depressive heterogeneity was reflected more clearly in reproducible local associations involving external factors within a joint depressive symptom-biomarker network than in broad network-wide differences. Associations involving sex formed the most stable biomarker-oriented pattern, and depressed mood consistently showed the highest symptom centrality across cohorts. These findings suggest a symptom-focused and biomarker-informed direction for assessment, while remaining exploratory group-level patterns rather than evidence for causal mechanisms, treatment selection, or individualized prediction. Future longitudinal and interventional studies with more detailed cardiovascular phenotyping are needed to determine whether these network patterns can inform personalized assessment or care.

## Data Availability

The CHARLS data analyzed in this study are publicly available from the official CHARLS repository. The independent hospital cohort data are not publicly available because they contain potentially identifiable participant information and are available from the corresponding authors on reasonable request, subject to institutional and ethical approval.
