# Supplementary material for: External factors show reproducible local symptom-biomarker associations in middle-aged and older adults with heart disease
Source: Front Psychiatry. 2026 Jun 2;17:1870992. doi: 10.3389/fpsyt.2026.1870992 (PMC13269108; doi:10.3389/fpsyt.2026.1870992)
Supplement: Supplementary file 1 [file Table1.docx]

**Supplementary Table S1.** Comparison between included and excluded participants in the discovery cohort

| **Characteristic** | **Included in discovery cohort (n = 1,685)** | **Excluded from discovery cohort (n = 1,170)** | **P value** |
| --- | --- | --- | --- |
| Age, years | 63.0 [56.0, 69.0] (n=1685) | 66.0 [59.0, 74.0] (n=171) | <0.001 |
| BMI, kg/m² | 24.8 [22.1, 27.5] (n=1685) | 23.9 [21.2, 26.7] (n=614) | <0.001 |
| **Sex** |  |  | 0.228 |
| Male | 679/1685 (40.3) | 444/1169 (38.0) |  |
| Female | 1006/1685 (59.7) | 725/1169 (62.0) |  |
| **Education level** |  |  | <0.001 |
| Primary school or below | 1435/1685 (85.2) | 172/305 (56.4) |  |
| Middle/High school | 209/1685 (12.4) | 105/305 (34.4) |  |
| College degree or above | 41/1685 (2.4) | 28/305 (9.2) |  |
| **Ever engaged in agricultural work** |  |  | <0.001 |
| Yes | 687/1685 (40.8) | 357/1162 (30.7) |  |
| No | 998/1685 (59.2) | 805/1162 (69.3) |  |
| **Marital status** |  |  | <0.001 |
| In a committed relationship | 1431/1685 (84.9) | 909/1170 (77.7) |  |
| Otherwise | 254/1685 (15.1) | 261/1170 (22.3) |  |

| **Characteristic** | **Included in discovery cohort (n = 1,685)** | **Excluded from discovery cohort (n = 1,170)** | **P value** |
| --- | --- | --- | --- |
| **Caregiver present** |  |  | <0.001 |
| Yes | 449/1685 (26.6) | 431/1170 (36.8) |  |
| No | 1236/1685 (73.4) | 739/1170 (63.2) |  |
| **Ever smoked** |  |  | 0.238 |
| Yes | 664/1685 (39.4) | 404/1089 (37.1) |  |
| No | 1021/1685 (60.6) | 685/1089 (62.9) |  |
| **Ever drank alcohol** |  |  | 0.010 |
| Yes | 493/1685 (29.3) | 290/1168 (24.8) |  |
| No | 1192/1685 (70.7) | 878/1168 (75.2) |  |

*Note.* Among 2,855 participants with physician-diagnosed heart disease identified in CHARLS 2015, 1,685 had complete data on all CES-D-10 items, the nine biomarkers, and the three external factors and were included in the final discovery cohort. CES-D-10, 10-item Center for Epidemiologic Studies Depression Scale; BMI, body mass index.

**Fig. S1** Flowchart of sample selection for the CHARLS discovery cohort


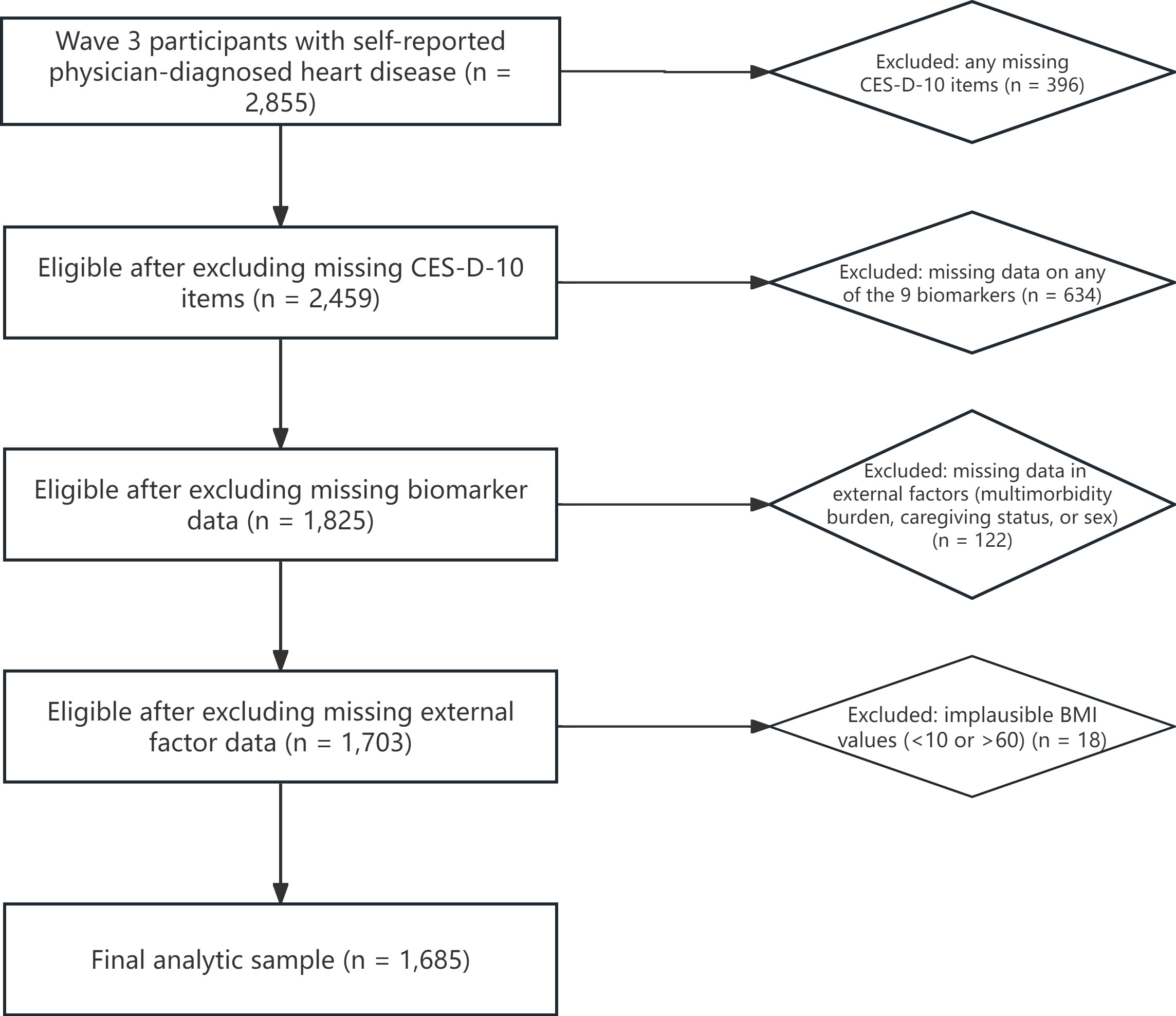


*Note.* The final analytic sample included 1,685 participants. CES-D-10, 10-item Center for Epidemiologic Studies Depression Scale; BMI, body mass index.
