# Supplementary material for: External factors show reproducible local symptom-biomarker associations in middle-aged and older adults with heart disease
Source: Front Psychiatry. 2026 Jun 2;17:1870992. doi: 10.3389/fpsyt.2026.1870992 (PMC13269108; doi:10.3389/fpsyt.2026.1870992)
Supplement: Supplementary file 2 [file Table2.docx]

| **Code** | **Variable** | **Type** | **Symptom/marker** | **Cluster** |
| --- | --- | --- | --- | --- |
| A1 | cesd1 | Depressive symptom | Bothered by small things | A Affective/interpersonal |
| B1 | cesd2 | Depressive symptom | Trouble concentrating | B Cognitive–somatic |
| A2 | cesd3 | Depressive symptom | Depressed mood | A Affective/interpersonal |
| B2 | cesd4 | Depressive symptom | Everything felt like an effort | B Cognitive–somatic |
| A3 | cesd5 | Depressive symptom | Lack of hope about the future | A Affective/interpersonal |
| A4 | cesd6 | Depressive symptom | Feeling fearful | A Affective/interpersonal |
| B3 | cesd7 | Depressive symptom | Restless sleep | B Cognitive–somatic |
| A5 | cesd8 | Depressive symptom | Unhappy | A Affective/interpersonal |
| A6 | cesd9 | Depressive symptom | Lonely | A Affective/interpersonal |
| B4 | cesd10 | Depressive symptom | Could not get going | B Cognitive–somatic |
| BMI | bmi_z | Biomarker | Body mass index (z score) | C Metabolic |
| SBP | sbp_mean_z | Biomarker | Mean systolic blood pressure (z) | C Metabolic |
| WBC | bl_wbc_z | Biomarker | White blood cell count (z) | D Inflammatory/renal |
| HDL | bl_hdl_z | Biomarker | High-density lipoprotein cholesterol (z) | C Metabolic |
| GLU | bl_glu_z | Biomarker | Fasting glucose (z) | C Metabolic |
| CysC | bl_cysc_z | Biomarker | Cystatin C (z) | D Inflammatory/renal |
| HbA1c | bl_hbalc_z | Biomarker | HbA1c (z) | C Metabolic |
| TG | bl_tg_log_z | Biomarker | Triglycerides (log z) | C Metabolic |
| CRP | bl_crp_log_z | Biomarker | C-reactive protein (log z) | D Inflammatory/renal |

**Supplementary Table S2.** Codes and descriptions of the 10 CES-D items and 9 cardiovascular/metabolic biomarkers.

*Note.* A1–A6 denote affective/interpersonal depressive symptoms; B1–B4 denote cognitive–somatic/behavioral symptoms. BMI, SBP, WBC, HDL-C, glucose, cystatin C, HbA1c, triglycerides, and CRP denote cardiovascular and metabolic biomarkers included in the network.
