# Supplementary material for: External factors show reproducible local symptom-biomarker associations in middle-aged and older adults with heart disease
Source: Front Psychiatry. 2026 Jun 2;17:1870992. doi: 10.3389/fpsyt.2026.1870992 (PMC13269108; doi:10.3389/fpsyt.2026.1870992)
Supplement: Supplementary file 3 [file Table3.docx]

**Supplementary Table S3.** Missingness across variables in the discovery and validation cohorts

*Discovery candidate sample before complete-case selection: n = 2,855; validation cohort before imputation: n = 506*

| **Domain** | **Variable** | **Discovery cohort missing n (%)** | **Validation cohort missing n (%)** |
| --- | --- | --- | --- |
| **Symptoms** |  |  |  |
|  | A1. Bothered by small things | 199 (7.0) | 0 (0.0) |
|  | A2. Depressed mood | 204 (7.1) | 0 (0.0) |
|  | A3. Lack of hope about the future | 283 (9.9) | 0 (0.0) |
|  | A4. Feeling fearful | 178 (6.2) | 0 (0.0) |
|  | A5. Unhappy | 195 (6.8) | 0 (0.0) |
|  | A6. Lonely | 194 (6.8) | 0 (0.0) |
|  | B1. Trouble concentrating | 242 (8.5) | 0 (0.0) |
|  | B2. Everything felt like an effort | 207 (7.3) | 0 (0.0) |
|  | B3. Restless sleep | 173 (6.1) | 0 (0.0) |
|  | B4. Could not get going | 209 (7.3) | 0 (0.0) |
| **Biomarkers** |  |  |  |
|  | BMI | 553 (19.4) | 0 (0.0) |
|  | Mean systolic blood pressure | 561 (19.6) | 0 (0.0) |
|  | WBC | 897 (31.4) | 0 (0.0) |
|  | HDL-C | 886 (31.0) | 0 (0.0) |
|  | Fasting glucose | 883 (30.9) | 0 (0.0) |
|  | Cystatin C | 887 (31.1) | 11 (2.2) |
|  | HbA1c | 879 (30.8) | 10 (2.0) |
|  | Triglycerides | 886 (31.0) | 0 (0.0) |
|  | CRP | 886 (31.0) | 0 (0.0) |
| **External factors** |  |  |  |
|  | Multimorbidity burden | 0 (0.0) | 0 (0.0) |
|  | Caregiving status | 0 (0.0) | 0 (0.0) |
|  | Sex | 1 (0.0) | 0 (0.0) |
| **Descriptive covariates** |  |  |  |
|  | Age | 999 (35.0) | 0 (0.0) |
|  | Education level | 865 (30.3) | 0 (0.0) |
|  | Marital status | 0 (0.0) | 0 (0.0) |
|  | Ever engaged in agricultural work | 8 (0.3) | 0 (0.0) |
|  | Ever smoked | 81 (2.8) | 0 (0.0) |
|  | Ever drank alcohol | 2 (0.1) | 0 (0.0) |

*Note.* Discovery missingness was calculated in the archived CHARLS heart-disease candidate sample before complete-case selection; validation missingness was calculated in the hospital cohort before imputation. Percentages are based on the cohort-specific denominator. In the main analysis, discovery-cohort missingness was handled by complete-case exclusion. In the validation cohort, only Cystatin C and HbA1c were multiply imputed in the primary analysis; all other listed variables were complete before imputation. Mean systolic blood pressure in the discovery cohort was derived from the available seated systolic measurements used in the analytic dataset.
