# Supplementary material for: External factors show reproducible local symptom-biomarker associations in middle-aged and older adults with heart disease
Source: Front Psychiatry. 2026 Jun 2;17:1870992. doi: 10.3389/fpsyt.2026.1870992 (PMC13269108; doi:10.3389/fpsyt.2026.1870992)
Supplement: Supplementary file 4 [file Table4.docx]

**Supplementary Table S4.** Node predictability in the 19-node depressive symptom–biomarker networks of the discovery and independent hospital cohorts

| **Code** | **Node** | **Domain** | **Cluster** | **Discovery cohort** | | | **Validation cohort, MI** | | | **Validation cohort, CC** | | |
| --- | --- | --- | --- | --- | --- | --- | --- | --- | --- | --- | --- | --- |
|  |  |  |  | **Neighbors** | **R²** | **Rank** | **Neighbors** | **R²** | **Rank** | **Neighbors** | **R²** | **Rank** |
| A1 | Bothered by small things | Depressive symptom | Affective/interpersonal | 8 | 0.413 | 2 | 4 | 0.401 | 3 | 5 | 0.413 | 3 |
| B1 | Trouble concentrating | Depressive symptom | Cognitive-somatic | 4 | 0.308 | 7 | 6 | 0.336 | 8 | 6 | 0.342 | 7 |
| A2 | Depressed mood | Depressive symptom | Affective/interpersonal | 7 | 0.491 | 1 | 5 | 0.468 | 1 | 5 | 0.468 | 1 |
| B2 | Everything felt like an effort | Depressive symptom | Cognitive-somatic | 6 | 0.393 | 3 | 6 | 0.382 | 5 | 5 | 0.386 | 5 |
| A3 | Lack of hope about the future | Depressive symptom | Affective/interpersonal | 2 | 0.200 | 13 | 1 | 0.193 | 11 | 1 | 0.194 | 12 |
| A4 | Feeling fearful | Depressive symptom | Affective/interpersonal | 6 | 0.283 | 9 | 4 | 0.189 | 12 | 4 | 0.198 | 11 |
| B3 | Restless sleep | Depressive symptom | Cognitive-somatic | 6 | 0.169 | 16 | 4 | 0.186 | 13 | 4 | 0.188 | 13 |
| A5 | Unhappy | Depressive symptom | Affective/interpersonal | 6 | 0.317 | 6 | 5 | 0.354 | 6 | 6 | 0.357 | 6 |
| A6 | Lonely | Depressive symptom | Affective/interpersonal | 7 | 0.352 | 5 | 5 | 0.315 | 9 | 5 | 0.312 | 9 |
| B4 | Could not get going | Depressive symptom | Cognitive-somatic | 7 | 0.371 | 4 | 7 | 0.344 | 7 | 6 | 0.339 | 8 |
| BMI | Body mass index | Biomarker | Metabolic biomarker | 7 | 0.187 | 15 | 5 | 0.163 | 15 | 5 | 0.170 | 15 |
| SBP | Mean systolic blood pressure | Biomarker | Metabolic biomarker | 3 | 0.036 | 19 | 1 | 0.018 | 19 | 1 | 0.022 | 19 |
| WBC | White blood cell count | Biomarker | Inflammatory/renal biomarker | 5 | 0.122 | 17 | 1 | 0.069 | 17 | 2 | 0.087 | 17 |
| HDL | High-density lipoprotein cholesterol | Biomarker | Metabolic biomarker | 4 | 0.190 | 14 | 4 | 0.396 | 4 | 4 | 0.393 | 4 |
| GLU | Fasting glucose | Biomarker | Metabolic biomarker | 3 | 0.252 | 10 | 2 | 0.213 | 10 | 2 | 0.206 | 10 |
| CysC | Cystatin C | Biomarker | Inflammatory/renal biomarker | 7 | 0.077 | 18 | 2 | 0.063 | 18 | 2 | 0.068 | 18 |
| HbA1c | Glycated hemoglobin | Biomarker | Metabolic biomarker | 4 | 0.237 | 11 | 2 | 0.128 | 16 | 2 | 0.111 | 16 |
| TG | Triglycerides | Biomarker | Metabolic biomarker | 4 | 0.289 | 8 | 3 | 0.422 | 2 | 4 | 0.427 | 2 |
| CRP | C-reactive protein | Biomarker | Inflammatory/renal biomarker | 4 | 0.211 | 12 | 5 | 0.173 | 14 | 5 | 0.174 | 14 |

*Note.* Predictability was quantified as the nodewise R², representing the proportion of variance in each node explained by its neighboring nodes in the estimated 19-node network. Discovery refers to the CHARLS 2015 cohort. Validation MI refers to the primary multiple-imputation analysis in the independent hospital cohort. Validation CC refers to the complete-case sensitivity analysis. Higher R² values indicate that a node is more strongly statistically accounted for by its neighboring nodes. Predictability was interpreted descriptively and should not be taken as evidence of causal controllability, temporal precedence, or intervention effects.
