# Supplementary material for: External factors show reproducible local symptom-biomarker associations in middle-aged and older adults with heart disease
Source: Front Psychiatry. 2026 Jun 2;17:1870992. doi: 10.3389/fpsyt.2026.1870992 (PMC13269108; doi:10.3389/fpsyt.2026.1870992)
Supplement: Supplementary file 5 [file Table5.docx]

**Supplementary Table S5.** Raw and analytical sample sizes for subgroup network comparison analyses in the CHARLS discovery cohort

| **Comparison** | **Group** | **Raw n** | **Analytical n** |
| --- | --- | --- | --- |
| **Main complete-case sample** | All included participants | 1,685 | 1,685 |
| **MM distribution in main complete-case sample** | MM 0–1 | 439 | 439 |
|  | MM 2–3 | 749 | 749 |
|  | MM ≥4 | 497 | 497 |
| **MM primary comparison** | Low MM (0–1) | 439 | 439 |
|  | High MM (≥4) | 497 | 497 |
| **Sex comparison after random subsampling** | Male | 679 | 679 |
|  | Female | 1,006 | 679 |
| **CG comparison after random subsampling** | Caregiver present (CG = 0) | 449 | 449 |
|  | Caregiver absent (CG = 1) | 1,236 | 449 |

*Note.* Multimorbidity (MM) was defined as the number of chronic conditions other than heart disease. For the primary MM comparison, the low-burden subgroup was defined as 0–1 conditions and the high-burden subgroup as ≥4 conditions; individuals with 2–3 conditions were excluded from this comparison by design. Caregiving status (CG) was derived from the available CHARLS caregiver item and reverse-coded for analysis, such that CG = 0 indicated caregiver presence and CG = 1 indicated caregiver absence. In the subgroup comparisons for sex and caregiving status, random subsampling was performed to balance group sizes before network comparison (seed = 123). Raw n denotes the subgroup size before subsampling, and analytical n denotes the final sample size used in the network comparison analysis.
