# Supplementary material for: External factors show reproducible local symptom-biomarker associations in middle-aged and older adults with heart disease
Source: Front Psychiatry. 2026 Jun 2;17:1870992. doi: 10.3389/fpsyt.2026.1870992 (PMC13269108; doi:10.3389/fpsyt.2026.1870992)
Supplement: Supplementary file 6 [file Table6.docx]

**Supplementary Table S6.** Descriptive characteristics of the CHARLS discovery cohort and the independent hospital cohort after preprocessing.

| **Characteristics** | **Discovery cohort (CHARLS) (N = 1,685)** | **Independent hospital cohort (N = 506)** |
| --- | --- | --- |
| Age, years | 63.00 [56.00, 69.00] | 63.00 [56.00, 70.00] |
| BMI, kg/m² | 24.83 [22.14, 27.48] | 24.25 [21.56, 27.00] |
| **Depressive symptoms** |  |  |
| CES-D-10 score | 8.00 [4.00, 14.00] | 10.00 [5.00, 16.00] |
| **CES-D-10 ≥10, n (%)** |  |  |
| Yes | 704 (41.8) | 263 (52.0) |
| No | 981 (58.2) | 243 (48.0) |
| **Sex** |  |  |
| Male | 679 (40.3) | 201 (39.7) |
| Female | 1006 (59.7) | 305 (60.3) |
| **Education level** |  |  |
| Primary school or below | 1435 (85.2) | 359 (70.9) |
| Middle/High school | 209 (12.4) | 132 (26.1) |
| College degree or above | 41 (2.4) | 15 (3.0) |
| **History of agricultural work** |  |  |
| Yes | 687 (40.8) | 220 (43.5) |
| No | 998 (59.2) | 286 (56.5) |
| **Relationship status** |  |  |
| In a committed relationship | 1431 (84.9) | 418 (82.6) |
| Otherwise | 254 (15.1) | 88 (17.4) |
| **Multimorbidity burden, n (%)** |  |  |
| Low | 439 (26.1) | 173 (34.2) |
| Medium | 749 (44.5) | 235 (46.4) |
| High | 497 (29.5) | 98 (19.4) |
| **Caregiving status, n (%)** |  |  |
| Caregiver absent | 1236 (73.4) | 401 (79.2) |
| Caregiver present | 449 (26.6) | 105 (20.8) |
| **Ever smoked** |  |  |
| Yes | 664 (39.4) | 188 (37.2) |
| No | 1021 (60.6) | 318 (62.8) |
| **Ever drank alcohol** |  |  |
| Yes | 493 (29.3) | 114 (22.5) |
| No | 1192 (70.7) | 392 (77.5) |

*Note.* Continuous variables are presented as median [Q1, Q3], and categorical variables as n (%). BMI, body mass index; CES-D-10, 10-item Center for Epidemiologic Studies Depression Scale.
