# Supplementary material for: External factors show reproducible local symptom-biomarker associations in middle-aged and older adults with heart disease
Source: Front Psychiatry. 2026 Jun 2;17:1870992. doi: 10.3389/fpsyt.2026.1870992 (PMC13269108; doi:10.3389/fpsyt.2026.1870992)
Supplement: Supplementary file 7 [file Table7.docx]

**Supplementary Table S7.** Strength and expected influence values for nodes in the 19-node discovery network

| **Code** | **Node** | **Cluster** | **Strength** | **Expected influence** |
| --- | --- | --- | --- | --- |
| A1 | Bothered by small things | Affective/interpersonal | 0.863 | 0.863 |
| B1 | Trouble concentrating | Cognitive–somatic | 0.512 | 0.512 |
| A2 | Depressed mood | Affective/interpersonal | 1.035 | 1.035 |
| B2 | Everything felt like an effort | Cognitive–somatic | 0.729 | 0.729 |
| A3 | Lack of hope about the future | Affective/interpersonal | 0.368 | 0.368 |
| A4 | Feeling fearful | Affective/interpersonal | 0.611 | 0.611 |
| B3 | Restless sleep | Cognitive–somatic | 0.322 | 0.322 |
| A5 | Unhappy | Affective/interpersonal | 0.705 | 0.705 |
| A6 | Lonely | Affective/interpersonal | 0.752 | 0.752 |
| B4 | Could not get going | Cognitive–somatic | 0.755 | 0.755 |
| BMI | Body mass index (BMI) | Metabolic biomarker | 0.454 | 0.122 |
| SBP | Mean systolic blood pressure (SBP) | Metabolic biomarker | 0.099 | 0.099 |
| WBC | White blood cell count (WBC) | Inflammatory/renal biomarker | 0.375 | 0.375 |
| HDL | High-density lipoprotein cholesterol (HDL-C) | Metabolic biomarker | 0.480 | -0.480 |
| GLU | Fasting glucose | Metabolic biomarker | 0.764 | 0.764 |
| CysC | Cystatin C | Inflammatory/renal biomarker | 0.260 | 0.086 |
| HbA1c | HbA1c | Metabolic biomarker | 0.759 | 0.759 |
| TG | Triglycerides (TG) | Metabolic biomarker | 0.711 | 0.203 |
| CRP | C-reactive protein (CRP) | Inflammatory/renal biomarker | 0.537 | 0.490 |

*Note:* Ordered to match Fig. 2B.
