# Supplementary material for: External factors show reproducible local symptom-biomarker associations in middle-aged and older adults with heart disease
Source: Front Psychiatry. 2026 Jun 2;17:1870992. doi: 10.3389/fpsyt.2026.1870992 (PMC13269108; doi:10.3389/fpsyt.2026.1870992)
Supplement: Supplementary file 8 [file Table8.docx]

**Supplementary Table S8.** External factor-node associations in the CHARLS discovery cohort under overall Bonferroni correction across 57 tests

| **External factor** | **Node code** | **Node** | **r** | **95% CI** | **Raw p** | **p_Bonf57** | **Significant** |
| --- | --- | --- | --- | --- | --- | --- | --- |
| Multimorbidity burden (MM) | A1 | Bothered by small things | -0.009 | [-0.050, 0.032] | 0.693 | 1.000 | No |
| Multimorbidity burden (MM) | B1 | Trouble concentrating | 0.015 | [-0.037, 0.065] | 0.511 | 1.000 | No |
| Multimorbidity burden (MM) | A2 | Depressed mood | 0.017 | [-0.024, 0.061] | 0.481 | 1.000 | No |
| Multimorbidity burden (MM) | B2 | Everything felt like an effort | 0.133 | [0.090, 0.174] | 8.74e-09 | 4.98e-07 | Yes |
| Multimorbidity burden (MM) | A3 | Lack of hope about the future | 0.030 | [-0.011, 0.078] | 0.193 | 1.000 | No |
| Multimorbidity burden (MM) | A4 | Feeling fearful | 0.005 | [-0.036, 0.054] | 0.833 | 1.000 | No |
| Multimorbidity burden (MM) | B3 | Restless sleep | 0.091 | [0.041, 0.131] | 0.0001 | 0.0063 | Yes |
| Multimorbidity burden (MM) | A5 | Unhappy | 0.065 | [0.014, 0.110] | 0.0054 | 0.308 | No |
| Multimorbidity burden (MM) | A6 | Lonely | 0.020 | [-0.023, 0.070] | 0.389 | 1.000 | No |
| Multimorbidity burden (MM) | B4 | Could not get going | 0.009 | [-0.041, 0.049] | 0.693 | 1.000 | No |
| Multimorbidity burden (MM) | BMI | Body mass index | 0.046 | [-0.001, 0.092] | 0.049 | 1.000 | No |
| Multimorbidity burden (MM) | SBP | Mean systolic blood pressure | 0.113 | [0.063, 0.154] | 1.17e-06 | 6.67e-05 | Yes |
| Multimorbidity burden (MM) | WBC | White blood cell count | 0.012 | [-0.035, 0.060] | 0.606 | 1.000 | No |
| Multimorbidity burden (MM) | HDL | High-density lipoprotein cholesterol | 0.004 | [-0.036, 0.043] | 0.873 | 1.000 | No |
| Multimorbidity burden (MM) | GLU | Fasting glucose | 0.016 | [-0.020, 0.061] | 0.475 | 1.000 | No |
| Multimorbidity burden (MM) | CysC | Cystatin C | 0.030 | [-0.023, 0.069] | 0.197 | 1.000 | No |
| Multimorbidity burden (MM) | HbA1c | Glycated hemoglobin | 0.090 | [0.051, 0.128] | 9.50e-05 | 0.0054 | Yes |
| Multimorbidity burden (MM) | TG | Triglycerides | 0.016 | [-0.029, 0.058] | 0.483 | 1.000 | No |
| Multimorbidity burden (MM) | CRP | C-reactive protein | 0.047 | [-0.001, 0.087] | 0.039 | 1.000 | No |
| Caregiving status (CG) | A1 | Bothered by small things | -0.031 | [-0.077, 0.019] | 0.205 | 1.000 | No |
| Caregiving status (CG) | B1 | Trouble concentrating | -0.034 | [-0.078, 0.011] | 0.152 | 1.000 | No |
| Caregiving status (CG) | A2 | Depressed mood | 0.016 | [-0.029, 0.066] | 0.505 | 1.000 | No |
| Caregiving status (CG) | B2 | Everything felt like an effort | -0.161 | [-0.207, -0.114] | 6.17e-12 | 3.52e-10 | Yes |
| Caregiving status (CG) | A3 | Lack of hope about the future | -0.023 | [-0.065, 0.016] | 0.330 | 1.000 | No |
| Caregiving status (CG) | A4 | Feeling fearful | 0.003 | [-0.048, 0.046] | 0.890 | 1.000 | No |
| Caregiving status (CG) | B3 | Restless sleep | -0.067 | [-0.120, -0.025] | 0.0048 | 0.275 | No |
| Caregiving status (CG) | A5 | Unhappy | -0.054 | [-0.094, -0.015] | 0.021 | 1.000 | No |
| Caregiving status (CG) | A6 | Lonely | -0.007 | [-0.052, 0.039] | 0.761 | 1.000 | No |
| Caregiving status (CG) | B4 | Could not get going | -0.035 | [-0.086, 0.012] | 0.146 | 1.000 | No |
| Caregiving status (CG) | BMI | Body mass index | -0.002 | [-0.042, 0.042] | 0.930 | 1.000 | No |
| Caregiving status (CG) | SBP | Mean systolic blood pressure | -0.066 | [-0.108, -0.024] | 0.0048 | 0.275 | No |
| Caregiving status (CG) | WBC | White blood cell count | -0.031 | [-0.075, 0.011] | 0.178 | 1.000 | No |
| Caregiving status (CG) | HDL | High-density lipoprotein cholesterol | -0.031 | [-0.075, 0.018] | 0.175 | 1.000 | No |
| Caregiving status (CG) | GLU | Fasting glucose | 0.050 | [0.008, 0.096] | 0.030 | 1.000 | No |
| Caregiving status (CG) | CysC | Cystatin C | -0.081 | [-0.124, -0.033] | 0.0005 | 0.030 | Yes |
| Caregiving status (CG) | HbA1c | Glycated hemoglobin | -0.030 | [-0.077, 0.012] | 0.189 | 1.000 | No |
| Caregiving status (CG) | TG | Triglycerides | -0.009 | [-0.059, 0.038] | 0.693 | 1.000 | No |
| Caregiving status (CG) | CRP | C-reactive protein | -0.019 | [-0.061, 0.020] | 0.400 | 1.000 | No |
| Sex | A1 | Bothered by small things | 0.011 | [-0.028, 0.061] | 0.641 | 1.000 | No |
| Sex | B1 | Trouble concentrating | 0.006 | [-0.040, 0.049] | 0.798 | 1.000 | No |
| Sex | A2 | Depressed mood | 0.039 | [-0.012, 0.082] | 0.090 | 1.000 | No |
| Sex | B2 | Everything felt like an effort | 0.035 | [-0.010, 0.074] | 0.121 | 1.000 | No |
| Sex | A3 | Lack of hope about the future | -0.021 | [-0.071, 0.026] | 0.347 | 1.000 | No |
| Sex | A4 | Feeling fearful | 0.052 | [0.009, 0.097] | 0.023 | 1.000 | No |
| Sex | B3 | Restless sleep | 0.143 | [0.094, 0.184] | 1.59e-10 | 9.04e-09 | Yes |
| Sex | A5 | Unhappy | -0.010 | [-0.053, 0.031] | 0.663 | 1.000 | No |
| Sex | A6 | Lonely | -0.015 | [-0.061, 0.026] | 0.516 | 1.000 | No |
| Sex | B4 | Could not get going | 0.007 | [-0.041, 0.049] | 0.744 | 1.000 | No |
| Sex | BMI | Body mass index | 0.109 | [0.066, 0.157] | 2.22e-06 | 0.0001 | Yes |
| Sex | SBP | Mean systolic blood pressure | -0.047 | [-0.089, -0.003] | 0.040 | 1.000 | No |
| Sex | WBC | White blood cell count | -0.141 | [-0.175, -0.106] | 3.56e-10 | 2.03e-08 | Yes |
| Sex | HDL | High-density lipoprotein cholesterol | 0.208 | [0.156, 0.251] | 2.69e-20 | 1.53e-18 | Yes |
| Sex | GLU | Fasting glucose | -0.023 | [-0.069, 0.023] | 0.309 | 1.000 | No |
| Sex | CysC | Cystatin C | -0.116 | [-0.160, -0.069] | 4.45e-07 | 2.53e-05 | Yes |
| Sex | HbA1c | Glycated hemoglobin | 0.072 | [0.028, 0.121] | 0.0015 | 0.087 | No |
| Sex | TG | Triglycerides | 0.176 | [0.126, 0.220] | 4.33e-15 | 2.47e-13 | Yes |
| Sex | CRP | C-reactive protein | -0.005 | [-0.050, 0.040] | 0.820 | 1.000 | No |

Note. r values are conditional association coefficients between each external factor and each network node. The overall Bonferroni-corrected threshold was p < 0.05/57 = 8.77 × 10^-4. p_Bonf57 was calculated as min(raw p × 57, 1). Significant = Yes indicates raw p < 8.77 × 10^-4. CG, caregiving status; MM, multimorbidity burden; CI, confidence interval; CES-D-10, 10-item Center for Epidemiologic Studies Depression Scale; BMI, body mass index; SBP, mean systolic blood pressure; WBC, white blood cell count; HDL-C, high-density lipoprotein cholesterol; GLU, fasting glucose; CysC, cystatin C; HbA1c, glycated hemoglobin; TG, triglycerides; CRP, C-reactive protein.
