# Supplementary material for: External factors show reproducible local symptom-biomarker associations in middle-aged and older adults with heart disease
Source: Front Psychiatry. 2026 Jun 2;17:1870992. doi: 10.3389/fpsyt.2026.1870992 (PMC13269108; doi:10.3389/fpsyt.2026.1870992)
Supplement: Supplementary file 9 [file Table9.docx]

**Supplementary Table S9.** Summary of moderated network model analyses across gamma settings in the discovery sample

| **Moderator** | **Gamma** | **n** | **Edges tested** | **Significant moderation effects, CI-based n** | **Significant moderation effects, strict n** |
| --- | --- | --- | --- | --- | --- |
| Multimorbidity burden (MM) | 0.00 | 1685 | 45 | 0 | 0 |
| Sex | 0.00 | 1685 | 45 | 0 | 0 |
| Caregiving status (CG) | 0.00 | 1685 | 45 | 0 | 0 |
| Multimorbidity burden (MM) | 0.10 | 1685 | 45 | 0 | 0 |
| Sex | 0.10 | 1685 | 45 | 0 | 0 |
| Caregiving status (CG) | 0.10 | 1685 | 45 | 0 | 0 |
| Multimorbidity burden (MM) | 0.25 | 1685 | 45 | 0 | 0 |
| Sex | 0.25 | 1685 | 45 | 0 | 0 |
| Caregiving status (CG) | 0.25 | 1685 | 45 | 0 | 0 |

*Note.* MNM = moderated network model. Gamma values of 0, 0.1, and 0.25 were examined as sensitivity settings. “Edges tested” refers to the 45 pairwise interactions among the 10 CES-D-10 symptom nodes. “CI-based” indicates moderation effects whose bootstrap confidence intervals excluded zero. “Strict” indicates moderation effects meeting the stricter predefined criterion in the analysis pipeline. No significant moderation effects were identified under any moderator or gamma setting.
