# Supplementary material for: External factors show reproducible local symptom-biomarker associations in middle-aged and older adults with heart disease
Source: Front Psychiatry. 2026 Jun 2;17:1870992. doi: 10.3389/fpsyt.2026.1870992 (PMC13269108; doi:10.3389/fpsyt.2026.1870992)
Supplement: Supplementary file 10 [file Table10.docx]

**Supplementary Table S10.** Differences in CES-D total scores, initial NCT results, and PSM trigger status across subgroup comparisons

| **Comparison** | **n_0_** | **n_1_** | **CES-D total, group 0** | **CES-D total, group 1** | **p (t-test)** | **Cohen's d** | **p (NCT structure)** | **p (global strength)** | **PSM** |
| --- | --- | --- | --- | --- | --- | --- | --- | --- | --- |
| MM: 0–1 vs ≥4 | 439 | 497 | 7.14 ± 5.63 | 12.00 ± 7.52 | 1.27e-27 | 0.725 | 0.658 | 0.228 | No |
| CG: caregiver absent (CG = 1) vs caregiver present (CG = 0) | 1236 | 449 | 8.35 ± 6.49 | 12.88 ± 7.31 | 1.65e-28 | 0.674 | 0.863 | 0.171 | No |
| Sex: male vs female | 679 | 1006 | 7.96 ± 6.30 | 10.64 ± 7.26 | 1.72e-15 | 0.389 | 0.184 | 0.543 | No |

*Note.* CES-D total differences were assessed using independent-samples t-tests in the base subgroup comparisons. Initial NCT results were based on 2,000 permutations. PSM was prespecified to be triggered only when both the CES-D total difference and the initial Network Comparison Test (NCT) network-structure test were significant. For presentation, n_0_ and n_1_ refer to the first and second subgroups listed in each row. MM = multimorbidity excluding heart disease; CG = caregiving status, coded as 0 = caregiver present and 1 = caregiver absent; PSM = propensity score matching; NCT = Network Comparison Test.
