# Supplementary material for: External factors show reproducible local symptom-biomarker associations in middle-aged and older adults with heart disease
Source: Front Psychiatry. 2026 Jun 2;17:1870992. doi: 10.3389/fpsyt.2026.1870992 (PMC13269108; doi:10.3389/fpsyt.2026.1870992)
Supplement: Supplementary file 11 [file Table11.docx]

**Supplementary Table S11.** Centrality metrics for the full 22-node mixed graphical model network in the CHARLS discovery cohort

| **Code** | **Node** | **Strength** | **z-Strength** | **Signed expected influence** | **z-EI** |
| --- | --- | --- | --- | --- | --- |
| A1 | Bothered by small things | 2.276 | 0.770 | 0.000 | -0.391 |
| A2 | Depressed mood | 3.165 | 1.694 | 0.000 | -0.391 |
| A3 | Lack of hope about the future | 1.300 | -0.246 | 0.000 | -0.391 |
| A4 | Feeling fearful | 2.985 | 1.507 | 0.000 | -0.391 |
| A5 | Unhappy | 2.509 | 1.011 | 0.000 | -0.391 |
| A6 | Lonely | 2.619 | 1.126 | 0.000 | -0.391 |
| B1 | Trouble concentrating | 1.716 | 0.187 | 0.000 | -0.391 |
| B2 | Everything felt like an effort | 2.952 | 1.472 | 0.000 | -0.391 |
| B3 | Restless sleep | 1.984 | 0.466 | 0.000 | -0.391 |
| B4 | Could not get going | 2.928 | 1.447 | 0.000 | -0.391 |
| BMI | Body mass index | 0.744 | -0.824 | 0.107 | -0.003 |
| SBP | Mean systolic blood pressure | 0.342 | -1.243 | 0.104 | -0.013 |
| HDL | High-density lipoprotein cholesterol | 0.833 | -0.732 | -0.499 | -2.196 |
| GLU | Fasting glucose | 0.913 | -0.648 | 0.779 | 2.427 |
| HbA1c | Glycated hemoglobin | 0.945 | -0.615 | 0.791 | 2.468 |
| TG | Triglycerides | 0.938 | -0.623 | 0.149 | 0.147 |
| WBC | White blood cell count | 0.615 | -0.958 | 0.328 | 0.795 |
| CysC | Cystatin C | 0.469 | -1.111 | 0.056 | -0.187 |
| CRP | C-reactive protein | 0.714 | -0.855 | 0.466 | 1.294 |
| MM | Multimorbidity burden excluding heart disease | 0.428 | -1.153 | 0.096 | -0.044 |
| Sex | Sex | 1.309 | -0.237 | 0.000 | -0.391 |
| CG | Caregiving status | 1.119 | -0.434 | 0.000 | -0.391 |

*Note.* Fig. 4B presents node strength only. Signed expected influence is reported here for completeness. Strength was defined as the sum of absolute edge weights, and signed expected influence as the sum of signed edge weights. MM was rank-transformed and z-standardized before network estimation. CG, caregiving status; EI, expected influence.
