# Supplementary material for: External factors show reproducible local symptom-biomarker associations in middle-aged and older adults with heart disease
Source: Front Psychiatry. 2026 Jun 2;17:1870992. doi: 10.3389/fpsyt.2026.1870992 (PMC13269108; doi:10.3389/fpsyt.2026.1870992)
Supplement: Supplementary file 12 [file Table12.docx]

**Supplementary Table S12.** Replication of core nodes and key edges in the independent hospital cohort

**Panel A. Replication of selected core nodes**

| **Domain** | **Node** | **Discovery rank (Strength / EI)** | **Validation rank (Strength / EI)** | **Validation CC rank (Strength / EI)** |
| --- | --- | --- | --- | --- |
| Symptom | A2 (Depressed mood) | 1 / 1 | 1 / 1 | 1 / 1 |
| Symptom | A5 (Unhappy) | 9 / 8 | 2 / 2 | 3 / 2 |
| Symptom | B4 (Could not get going) | 5 / 5 | 3 / 6 | 4 / 6 |
| Symptom | A1 (Bothered) | 2 / 2 | 9 / 4 | 5 / 3 |
| Symptom | A6 (Lonely) | 6 / 6 | 10 / 5 | 9 / 5 |
| Biomarker | TG (Triglycerides) | 8 / 15 | 4 / 18 | 2 / 18 |
| Biomarker | HDL-C (HDL-C) | 13 / 19 | 7 / 19 | 8 / 19 |
| Biomarker | CRP (CRP) | 11 / 11 | 8 / 11 | 10 / 11 |

**Panel B. Replication of key edges**

| **Edge** | **Discovery weight** | **Validation weight** | **Validation abs-rank** | **Direction consistent** |
| --- | --- | --- | --- | --- |
| A3–A5 (Lack of hope–Unhappy) | 0.410 | 0.388 | 2 | Yes |
| A1–A2 (Bothered–Depressed mood) | 0.308 | 0.338 | 3 | Yes |
| HDL-C–Triglycerides | -0.282 | -0.530 | 1 | Yes |
| Glucose–HbA1c | 0.403 | 0.311 | 4 | Yes |
| WBC–CRP | 0.232 | 0.247 | 6 | Yes |

*Note.* Panel A reports selected symptom and biomarker nodes emphasized in the main Results. Ranks are shown as Strength / Expected Influence, with lower rank values indicating greater centrality. Validation CC refers to the complete-case sensitivity analysis in the validation cohort. Panel B reports key edges highlighted in the discovery sample and their corresponding edge weights in the validation cohort. Validation abs-rank indicates the rank of the absolute edge weight in the validation network. Edge replication was judged primarily by consistency in direction and relative prominence across cohorts.

**Fig. S2** Nineteen-node symptom–biomarker network in the independent hospital cohort.

| a. |
| --- |
|  |
| b. |
| 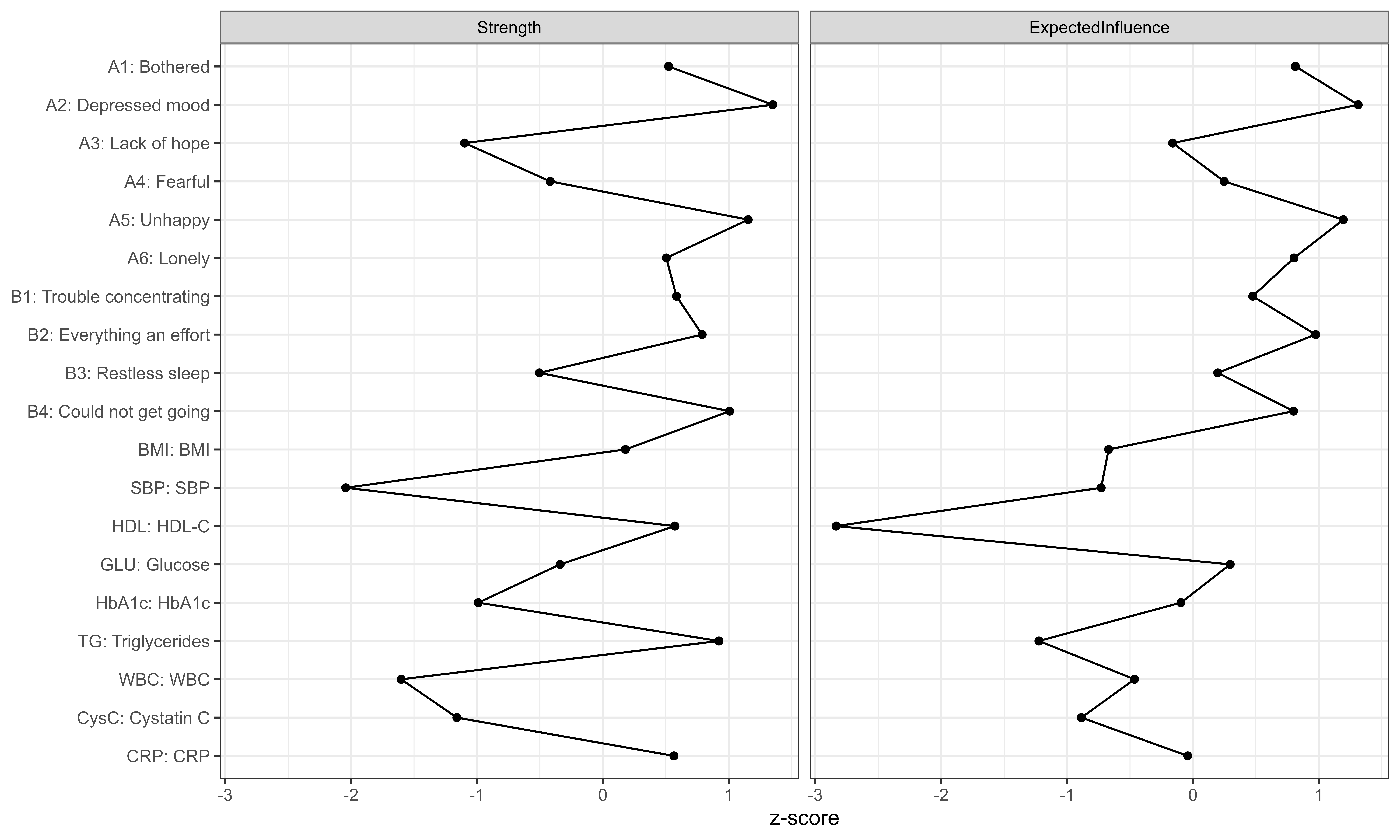 |

*Note.* a) Network of depressive symptoms and biomarkers in the independent hospital cohort. Depicted in the MDS graph layout, where the proximity between nodes was proportional to edge strength. Blue edges indicate positive relationships; red edges indicate negative relationships. Light blue nodes denote affective/interpersonal depressive symptoms; light green nodes denote cognitive–somatic depressive symptoms; tan nodes denote metabolic biomarkers; pink nodes denote inflammatory/renal biomarkers. b) Node centrality. The x-axis represents the z-score. Depressive symptoms: A1, Bothered by small things; A2, Depressed mood; A3, Lack of hope about the future; A4, Feeling fearful; A5, Unhappy; A6, Lonely; B1, Trouble concentrating; B2, Everything felt like an effort; B3, Restless sleep; B4, Could not get going. Biomarkers: BMI, body mass index; SBP, mean systolic blood pressure; WBC, white blood cell count; HDL, high-density lipoprotein cholesterol; GLU, fasting glucose; CysC, cystatin C; HbA1c, glycated hemoglobin; TG, triglycerides; CRP, C-reactive protein.

.
