# Supplementary material for: External factors show reproducible local symptom-biomarker associations in middle-aged and older adults with heart disease
Source: Front Psychiatry. 2026 Jun 2;17:1870992. doi: 10.3389/fpsyt.2026.1870992 (PMC13269108; doi:10.3389/fpsyt.2026.1870992)
Supplement: Supplementary file 13 [file Table13.docx]

**Supplementary Table S13.** External factor-node associations in the independent hospital cohort under overall Bonferroni correction across 57 tests

| **External factor** | **Node code** | **Node** | **r** | **95% CI** | **Raw p** | **p_Bonf57** | **Significant** |
| --- | --- | --- | --- | --- | --- | --- | --- |
| Multimorbidity burden (MM) | A1 | Bothered by small things | -0.045 | [-0.125, 0.028] | 0.285 | 1.000 | No |
| Multimorbidity burden (MM) | B1 | Trouble concentrating | 0.050 | [-0.032, 0.132] | 0.241 | 1.000 | No |
| Multimorbidity burden (MM) | A2 | Depressed mood | 0.068 | [-0.013, 0.153] | 0.105 | 1.000 | No |
| Multimorbidity burden (MM) | B2 | Everything felt like an effort | 0.093 | [0.007, 0.168] | 0.027 | 1.000 | No |
| Multimorbidity burden (MM) | A3 | Lack of hope about the future | 0.001 | [-0.085, 0.087] | 0.981 | 1.000 | No |
| Multimorbidity burden (MM) | A4 | Feeling fearful | 0.060 | [-0.018, 0.134] | 0.155 | 1.000 | No |
| Multimorbidity burden (MM) | B3 | Restless sleep | 0.117 | [0.033, 0.198] | 0.0054 | 0.309 | No |
| Multimorbidity burden (MM) | A5 | Unhappy | 0.059 | [-0.026, 0.137] | 0.157 | 1.000 | No |
| Multimorbidity burden (MM) | A6 | Lonely | 0.019 | [-0.057, 0.101] | 0.662 | 1.000 | No |
| Multimorbidity burden (MM) | B4 | Could not get going | -0.031 | [-0.115, 0.050] | 0.472 | 1.000 | No |
| Multimorbidity burden (MM) | BMI | Body mass index | 0.073 | [-0.011, 0.164] | 0.079 | 1.000 | No |
| Multimorbidity burden (MM) | SBP | Mean systolic blood pressure | 0.138 | [0.054, 0.209] | 0.0007 | 0.040 | Yes |
| Multimorbidity burden (MM) | WBC | White blood cell count | 0.060 | [-0.019, 0.142] | 0.138 | 1.000 | No |
| Multimorbidity burden (MM) | HDL | High-density lipoprotein cholesterol | 0.088 | [0.004, 0.161] | 0.033 | 1.000 | No |
| Multimorbidity burden (MM) | GLU | Fasting glucose | 0.029 | [-0.057, 0.111] | 0.480 | 1.000 | No |
| Multimorbidity burden (MM) | CysC | Cystatin C | 0.003 | [-0.078, 0.085] | 0.933 | 1.000 | No |
| Multimorbidity burden (MM) | HbA1c | Glycated hemoglobin | 0.068 | [-0.006, 0.149] | 0.099 | 1.000 | No |
| Multimorbidity burden (MM) | TG | Triglycerides | 0.114 | [0.032, 0.188] | 0.0054 | 0.310 | No |
| Multimorbidity burden (MM) | CRP | C-reactive protein | 0.040 | [-0.041, 0.120] | 0.333 | 1.000 | No |
| Caregiving status (CG) | A1 | Bothered by small things | 0.011 | [-0.070, 0.092] | 0.801 | 1.000 | No |
| Caregiving status (CG) | B1 | Trouble concentrating | -0.077 | [-0.170, 0.013] | 0.074 | 1.000 | No |
| Caregiving status (CG) | A2 | Depressed mood | -0.001 | [-0.092, 0.091] | 0.981 | 1.000 | No |
| Caregiving status (CG) | B2 | Everything felt like an effort | -0.124 | [-0.212, -0.031] | 0.0037 | 0.210 | No |
| Caregiving status (CG) | A3 | Lack of hope about the future | -0.085 | [-0.157, -0.009] | 0.041 | 1.000 | No |
| Caregiving status (CG) | A4 | Feeling fearful | 0.067 | [-0.033, 0.155] | 0.119 | 1.000 | No |
| Caregiving status (CG) | B3 | Restless sleep | -0.016 | [-0.095, 0.076] | 0.706 | 1.000 | No |
| Caregiving status (CG) | A5 | Unhappy | -0.008 | [-0.095, 0.079] | 0.849 | 1.000 | No |
| Caregiving status (CG) | A6 | Lonely | -0.026 | [-0.113, 0.064] | 0.542 | 1.000 | No |
| Caregiving status (CG) | B4 | Could not get going | -0.102 | [-0.199, -0.009] | 0.017 | 0.997 | No |
| Caregiving status (CG) | BMI | Body mass index | 0.101 | [0.013, 0.182] | 0.015 | 0.836 | No |
| Caregiving status (CG) | SBP | Mean systolic blood pressure | -0.061 | [-0.138, 0.024] | 0.141 | 1.000 | No |
| Caregiving status (CG) | WBC | White blood cell count | 0.010 | [-0.066, 0.086] | 0.809 | 1.000 | No |
| Caregiving status (CG) | HDL | High-density lipoprotein cholesterol | -0.041 | [-0.128, 0.050] | 0.328 | 1.000 | No |
| Caregiving status (CG) | GLU | Fasting glucose | 0.041 | [-0.044, 0.134] | 0.320 | 1.000 | No |
| Caregiving status (CG) | CysC | Cystatin C | -0.032 | [-0.117, 0.048] | 0.447 | 1.000 | No |
| Caregiving status (CG) | HbA1c | Glycated hemoglobin | -0.069 | [-0.147, 0.009] | 0.100 | 1.000 | No |
| Caregiving status (CG) | TG | Triglycerides | -0.094 | [-0.171, -0.006] | 0.022 | 1.000 | No |
| Caregiving status (CG) | CRP | C-reactive protein | -0.108 | [-0.183, -0.022] | 0.010 | 0.589 | No |
| Sex | A1 | Bothered by small things | 0.034 | [-0.054, 0.117] | 0.415 | 1.000 | No |
| Sex | B1 | Trouble concentrating | 0.016 | [-0.066, 0.092] | 0.703 | 1.000 | No |
| Sex | A2 | Depressed mood | 0.034 | [-0.046, 0.115] | 0.414 | 1.000 | No |
| Sex | B2 | Everything felt like an effort | -0.001 | [-0.082, 0.082] | 0.987 | 1.000 | No |
| Sex | A3 | Lack of hope about the future | -0.010 | [-0.088, 0.069] | 0.802 | 1.000 | No |
| Sex | A4 | Feeling fearful | 0.075 | [-0.007, 0.154] | 0.072 | 1.000 | No |
| Sex | B3 | Restless sleep | 0.124 | [0.037, 0.202] | 0.0027 | 0.151 | No |
| Sex | A5 | Unhappy | -0.013 | [-0.096, 0.061] | 0.744 | 1.000 | No |
| Sex | A6 | Lonely | 0.005 | [-0.067, 0.084] | 0.900 | 1.000 | No |
| Sex | B4 | Could not get going | -0.018 | [-0.098, 0.065] | 0.676 | 1.000 | No |
| Sex | BMI | Body mass index | 0.061 | [-0.023, 0.142] | 0.153 | 1.000 | No |
| Sex | SBP | Mean systolic blood pressure | 0.002 | [-0.073, 0.080] | 0.952 | 1.000 | No |
| Sex | WBC | White blood cell count | -0.089 | [-0.169, -0.012] | 0.029 | 1.000 | No |
| Sex | HDL | High-density lipoprotein cholesterol | 0.225 | [0.143, 0.297] | 2.57e-08 | 1.47e-06 | Yes |
| Sex | GLU | Fasting glucose | -0.035 | [-0.119, 0.050] | 0.399 | 1.000 | No |
| Sex | CysC | Cystatin C | -0.165 | [-0.238, -0.085] | 4.92e-05 | 0.0028 | Yes |
| Sex | HbA1c | Glycated hemoglobin | 0.000 | [-0.075, 0.083] | 0.999 | 1.000 | No |
| Sex | TG | Triglycerides | 0.236 | [0.156, 0.313] | 4.00e-09 | 2.28e-07 | Yes |
| Sex | CRP | C-reactive protein | 0.039 | [-0.045, 0.118] | 0.353 | 1.000 | No |

Note. r values are conditional association coefficients between each external factor and each network node. The overall Bonferroni-corrected threshold was p < 0.05/57 = 8.77 × 10^-4. p_Bonf57 was calculated as min(raw p × 57, 1). Significant = Yes indicates raw p < 8.77 × 10^-4. CG, caregiving status; MM, multimorbidity burden; CI, confidence interval; CES-D-10, 10-item Center for Epidemiologic Studies Depression Scale; BMI, body mass index; SBP, mean systolic blood pressure; WBC, white blood cell count; HDL-C, high-density lipoprotein cholesterol; GLU, fasting glucose; CysC, cystatin C; HbA1c, glycated hemoglobin; TG, triglycerides; CRP, C-reactive protein.
